# Natural Compounds' Activity against Cancer Stem-Like or Fast-Cycling Melanoma Cells

**DOI:** 10.1371/journal.pone.0090783

**Published:** 2014-03-03

**Authors:** Malgorzata Sztiller-Sikorska, Kamila Koprowska, Kinga Majchrzak, Mariusz Hartman, Malgorzata Czyz

**Affiliations:** Department of Molecular Biology of Cancer, Medical University of Lodz, Lodz, Poland; Spanish National Cancer Centre (CNIO), Spain

## Abstract

**Background:**

Accumulating evidence supports the concept that melanoma is highly heterogeneous and sustained by a small subpopulation of melanoma stem-like cells. Those cells are considered as responsible for tumor resistance to therapies. Moreover, melanoma cells are characterized by their high phenotypic plasticity. Consequently, both melanoma stem-like cells and their more differentiated progeny must be eradicated to achieve durable cure. By reevaluating compounds in heterogeneous melanoma populations, it might be possible to select compounds with activity not only against fast-cycling cells but also against cancer stem-like cells. Natural compounds were the focus of the present study.

**Methods:**

We analyzed 120 compounds from The Natural Products Set II to identify compounds active against melanoma populations grown in an anchorage-independent manner and enriched with cells exerting self-renewing capacity. Cell viability, cell cycle arrest, apoptosis, gene expression, clonogenic survival and label-retention were analyzed.

**Findings:**

Several compounds efficiently eradicated cells with clonogenic capacity and nanaomycin A, streptonigrin and toyocamycin were effective at 0.1 µM. Other anti-clonogenic but not highly cytotoxic compounds such as bryostatin 1, siomycin A, illudin M, michellamine B and pentoxifylline markedly reduced the frequency of ABCB5 (ATP-binding cassette, sub-family B, member 5)-positive cells. On the contrary, treatment with maytansine and colchicine selected for cells expressing this transporter. Maytansine, streptonigrin, toyocamycin and colchicine, even if highly cytotoxic, left a small subpopulation of slow-dividing cells unaffected. Compounds selected in the present study differentially altered the expression of melanocyte/melanoma specific microphthalmia-associated transcription factor (MITF) and proto-oncogene c-MYC.

**Conclusion:**

Selected anti-clonogenic compounds might be further investigated as potential adjuvants targeting melanoma stem-like cells in the combined anti-melanoma therapy, whereas selected cytotoxic but not anti-clonogenic compounds, which increased the frequency of ABCB5-positive cells and remained slow-cycling cells unaffected, might be considered as a tool to enrich cultures with cells exhibiting melanoma stem cell characteristics.

## Introduction

The intratumoral phenotypic heterogeneity results from the genetic variation but also from the plasticity of tumor cells that is observed in response to microenvironmental stimuli. Among diverse functional phenotypes within a tumor, a subpopulation of cancer stem-like cells (CSCs) capable of self-renewal and the bulk of a tumor consisting of fast-cycling cells and more differentiated cells could be distinguished [1]–[3]. As the phenotypic heterogeneity was shown to be highly dynamic in many tumors including melanoma [4]–[7] and the therapeutic eradication of CSC subpopulation may be followed by its regeneration from non-CSCs, both CSCs and the bulk population should be considered in developing the anticancer therapy [8]–[14]. Therefore, a drug combination causing a complete eradication of all kinds of cells within a tumor might be necessary to achieve durable cures. In the selection process of highly potent drug candidates, there is a substantial problem in creating experimental *in vitro* models that reliably predict drug activity in patients. In the present study, melanoma cells obtained directly from pathologically distinct specimens, nodular melanoma and superficial spreading melanoma, were grown in an anchorage-independent manner in stem cell medium and were enriched with cells exerting self-renewing capacity in comparison to serum-driven monolayers [15]. This *in vitro* three-dimensional model has also been shown to preserve the heterogeneity of the original tumor more accurately than two-dimensional monolayer cultures [16]–[18] and was an important element of novelty in the present *in vitro* screening of the natural compound library.

Natural compounds are widely used in anticancer therapy and can exert considerable biological activity [19]–[21]. Although their mechanisms of action are often not well defined, most of therapeutic agents derived from natural products are mainly effective at eliminating cancer cells with a high proliferation rate. Compounds that affect cell division may fail, however, to eradicate the subpopulation of slow-cycling cancer stem-like cells, leading eventually to tumor relapse. In the present study, although several approaches have been used in the selection process, priority was given to those compounds that were capable of reducing the number of clonogenic cells. A reduction in clonogenicity was interpreted as a direct effect on the self-renewing potential of the cancer stem-like cells. In addition, apoptosis, label-retention, frequency of ABCB5-positive cells and the expression of Wnt/β-catenin signaling target genes, *MITF* and *c-MYC*, were assessed in melanoma populations treated with compounds selected as either highly anti-clonogenic or highly cytotoxic. We investigated the influence of selected compounds on stemness-associated molecules and pathways. Slow-cycling cells considered as cancer stem-like cells (CSCs) can be marked as label-retaining cells [12]. In the present study, we used a fluorescent dye CFSE to identify compounds that left the label-retaining melanoma cells unaffected. ABCB5 transporter protein, a mediator of chemoresistance in melanoma, is also recognized as a potential marker for melanoma stem-like cells [2], [22]. Melanoma cells that express ABCB5 protein could selectively survive when exposed to dacarbazine, vemurafenib and other drugs [22]. It was demonstrated that anti-ABCB5 antibody or siRNA gene silencing reversed melanoma resistance to anticancer drugs [23], [24]. We investigated whether the proportion of cells carrying this transporter was altered upon the treatment with selected natural compounds. Wnt signaling plays a crucial role in preserving the pluripotency of embryonic stem cells and cancer development [25]. Recent report suggested a role for the Wnt/β-catenin signaling pathway in maintaining the viability and/or sustaining the self-renewal of breast tumor initiating cells *in vitro* [26]. In melanoma, β-catenin signaling increases during tumor progression and it promotes chemoresistance [27]. Our recent study revealed that the Wnt/β-catenin signaling pathway is suppressed in melanoma populations with the low frequency of clonogenic cells (Hartman et al., submitted). The influence of selected natural compounds on two Wnt/β-catenin target genes, *MITF* and *c-MYC* was investigated. Transcription factor MITF acts as a rheostat determining the phenotypic identity among different subpopulations of melanoma cells [28]. Although amplified only in about 20% of melanomas, MITF has been proposed as a lineage addiction oncogene contributing to melanoma chemoresistance [29], [30]. The proto-oncogene c-MYC plays a crucial role in the development of a large number of human tumors [31]. Most recently, it has been shown that over-expression of c-MYC within the tumor can be targeted with specific T-cells [32]. Compounds inducing apoptosis and/or inhibiting proliferation in a c-MYC-specific manner act on distinct cellular targets [33], [34]. c-MYC inhibition in melanoma cells results in reduced proliferation through mechanisms involving several enzymes of nucleotide biosynthesis [35].

To our knowledge, there are no previous studies exploring so many factors in parallel in the initial selection process for active compounds from The Natural Products Set II (NCI). Based on the created activity profiles, each of the selected compounds can be further investigated for its potential applicability as a part of anticancer therapy or as a tool in the laboratory work. The natural products selected as active compounds against melanoma cells can also provide novel leads for conversion into clinically useful agents.

## Materials and Methods

### Compounds

The Natural Products Set II was obtained from the US National Cancer Institute (NCI, http://www.dtp.nci.nih.gov). The compounds were in 96-well plates with 60 compounds per plate. Plates were stored dry at −20°C. Each well contained 0.02 µmol of compound in 1 µl of glycerol. 10 mM solution (20 µl) of each compound was obtained by the addition of 19 µl of DMSO to each well. Immediately after solubilization the drug solutions were aliqouted into several testing plates to avoid possible precipitation of the compound. All experiments were performed using compounds provided by NCI.

### Tumor Tissues and Ethics Statement

DMBC cells were obtained in Department of Molecular Biology of Cancer from surgical specimens of melanoma in advanced stages. Patient characteristics of melanoma specimens were published previously [15], [36], [37]. The study was approved by the Ethical Commission of the Medical University of Lodz and written informed consent was obtained from all patients.

### Cell Cultures

Cells were maintained in stem cells medium (SCM) in an anchorage-independent manner as described previously [36].

### Measurement of Viable Cell Number

Melanoma cells were counted after staining with Trypan blue (Sigma-Aldrich) and plated at a density of 4 × 10^3^ viable cells per well in 96-well plates. Cells were cultured for 45 h with vehicle (0.05% DMSO) or the compounds from The Natural Products Set II at 5 µM. An acid phosphatase activity (APA) assay was used to measure viable cell number. Briefly, the plates were centrifuged, the medium was discarded and replaced with 100 µl assay buffer containing 0.1 M sodium acetate (pH = 5), 0.1% Triton X-100 and 5 mM p-nitrophenyl phosphate, pNPP (Sigma-Aldrich) and incubated for additional 2 h at 37°C. The reaction was stopped with 10 µl/well of 1 M NaOH, and the absorbance values were measured at the wavelength of 405 nm using a microplate reader (Infinite M200Pro, Tecan, Austria). Melanoma cells did not respond to 0.05% DMSO with reduced viability.

### Viability Assay

Drug-induced changes in cell viability after 45 h treatment were also assessed by flow cytometry after propidium iodide (PI) staining or by using an automated cell viability analyzer according to standard procedures. In both assays, cells were analyzed using a FACSVerse flow cytometer (Becton Dickinson, San Jose, California, USA), and results were processed by using FACSuite software (Becton Dickinson).

### Cell Cycle Analysis

Melanoma cells were treated with the vehicle or the compounds at the indicated concentrations for 30 h or 45 h, then collected and fixed with 70% (w/v) ethanol at −20°C. Cells were washed twice with PBS and resuspended in PI Staining Buffer containing RNAse (Becton Dickinson, San Jose, CA, USA). Following incubation for 30 min at room temperature in the dark, cells were analyzed using a FACSVerse flow cytometer (Becton Dickinson). ModFit LT 3.0 software (Verify Software, Topsham, MN, USA) was used to calculate the percentages of cells in each cell cycle phase, and FACSuit software (Becton Dickinson) was used to calculate the percentages of dead cells in subG_1_.

### Clonogenic Assay

Melanoma cells were first incubated with compounds at indicated concentrations for 4 h. Then, viability was determined by Trypan blue staining and 1000 single, viable melanoma cells were transferred to 700 µl top agar medium mixture (SCM, 0.35% (w/v) agar) and the obtained cell suspensions were overlaid onto 12-well culture plates coated with 700 µl solidified bottom agar mixture (SCM, 0.5% (w/v) agar). The plates were then incubated at 37°C in a humidified incubator for 2 weeks, and in some cases also for 3 weeks. Cells were stained with 500 µl of 0.005% crystal violet for 2 h, and colonies at least 50 µm in diameter were counted under the microscope. The influence of the compounds on the clonogenicity was expressed as percent of control according to the formula: number colonies generated after treatment with the compounds/number of colonies in control with the vehicle × 100.

### Flow Cytometric Analysis of Apoptosis

Detection of cell death was carried out by dual staining with Annexin V-FITC and PI (Roche Diagnostics, Manheim, Germany). Melanoma cells were seeded into 12-well plates and treated for 45 h with the compounds at indicated concentrations. After treatment, cells were collected, centrifuged at 400×g for 5 min and stained with Annexin V-FITC and PI for 15 min at room temperature in the dark. 15,000 events were analyzed for each sample by flow cytometer FACSVerse (Becton Dickinson), and results were processed by using FACSuite software (Becton Dickinson).

### Assessment of ABCB5-Positive Cells

The frequency of cells expressing ABCB5 was assessed by flow cytometry. Unconjugated anti-ABCB5 primary antibody from Sigma-Aldrich and FITC-conjugated goat anti-rabbit secondary antibody (BD Pharmingen) were used in this study. Typically, 30,000 cells were analyzed per sample. Isotype controls were included in each experiment. To exclude dead cells from the analysis, 7-aminoactinomycin D staining (7-AAD; eBiosciences) was used. Flow cytometric acquisition was performed using a FACSVerse flow cytometer (Becton Dickinson) and analyzed using FACSuite software. Changes in the frequency of ABCB5-positive cells after drug treatment for 45 h were expressed as percentages of control with vehicle.

### CFSE Staining

For CFSE analysis, melanoma cells were stained with 1.5 µM CFSE (the prodrug carboxyfluorescein diacetate succinimidyl ester) for 30 min at 37°C, washed twice, seeded in twelve-well plates at 1.25×10^5^/well, and exposed to the compounds for 5 days at indicated concentrations. Then, medium was exchanged and cells were incubated in drug-free medium for additional 6 days, and assessed by flow cytometry. Green fluorescence emission was measured using a FACSVerse flow cytometer (Becton Dickinson) and analyzed using FACSuite software.

### RNA Isolation, cDNA Synthesis and Real-Time PCR

RNA was isolated and purified using Total RNA Isolation kit with mini column system (A&A Biotechnology, Gdynia, Poland). The RNA concentration and purity were measured with a Tecan NanoQuant Plate reader (Tecan, Austria). cDNA was synthesized by using 300 ng of random primers and SuperScript II Reverse Transcriptase (Invitrogen Life Technologies, Carlsbad, CA, USA). The Rotor-Gene 3000 Real-Time DNA analysis system (Corbett Research, Morklake, Australia) was used to evaluate the gene expression by quantitative real-time polymerase chain reaction (qRT-PCR). The amplification was performed by using KAPA SYBR FAST qPCR Kit Universal 2X qPCR Master Mix (Kapa Biosystems, Cape Town, South Africa), 200 nM of each primer and 25 ng cDNA template per reaction. The primers used for Real-Time PCR were as following: MITF: 5′-ACC GTC TCT CAC TGG ATT GG-3′ and 5′-TAC TTG GTG GGG TTT TCG AG-3′; C-MYC: 5′-AAT GAA AAG GCC CCC AAG GTA GTT ATC C-3′ and 5′-GTC GTT TCC GCA ACA AGT CCT CTT C-3′; SOX2: 5′-GCT AGT CTC CAA GCG ACG-3′ and 5′-GCA AGA AGC CTC TCC TTG-3′. The annealing temperature for all genes was 56°C. The relative expression of target genes was calculated versus a reference gene RPS17 (with primers: 5′-AAT CTC CTG ATC CAA GGC TG-3′ and 5′-CAA GAT AGC AGG TTA TGT CAC G-3′), and a mathematical model including an efficiency correction for Real-Time PCR was used.

### Statistical Analysis

Data represent means ± SD from three separate experiments unless otherwise specified. The significance of an apparent difference in mean values for any tested parameter was validated by a Student's paired t test. P<0.05 was considered significant.

## Results

### Selection Strategy of Natural Compounds in Melanoma Cells Grown in an Anchorage-Independent Manner

**Figure 1:**
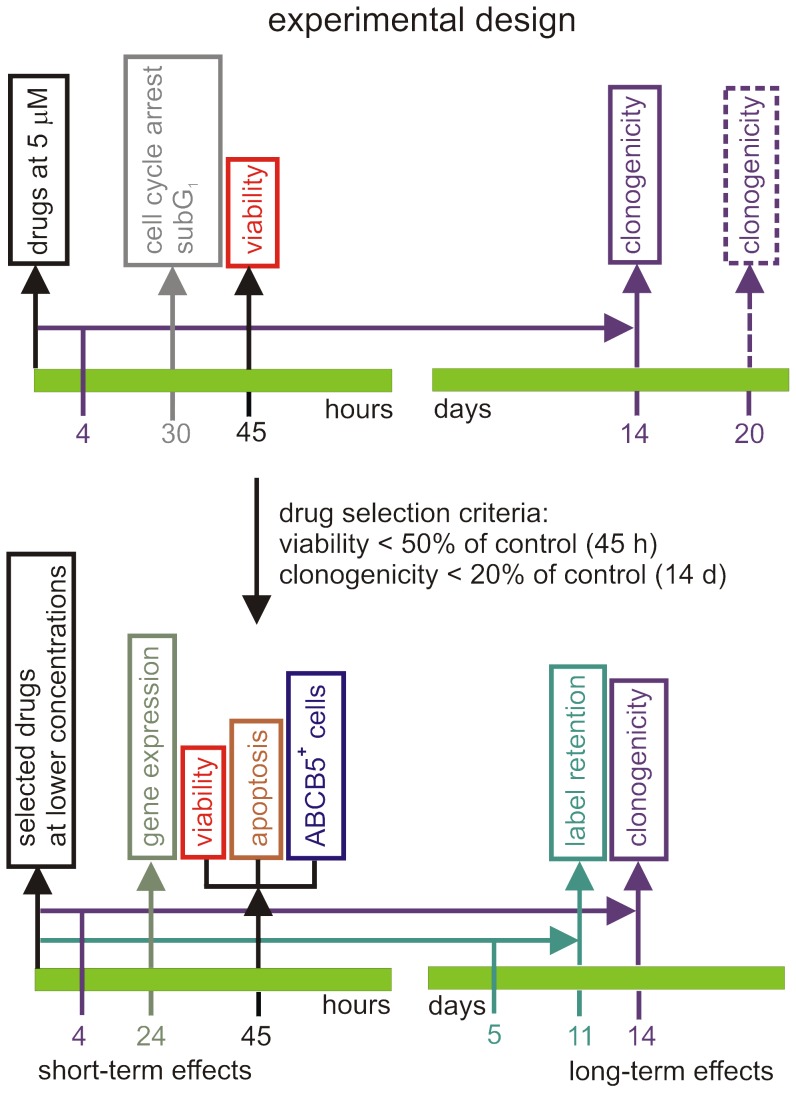
The compound screening procedure. First, compounds from The Natural Products Set II were tested in two melanoma cell lines obtained from pathologically distinct specimens, DMBC11 and DMBC12, at a single concentration of 5 µM. Changes in viability (APA assay, volumetric assay, PI staining and subG_1_ assay) were measured as short-term effects and changes in clonogenic potential as long-term effects of the tested compounds. All viability tests were compared for potential inconsistencies. Two criteria were used to select compounds for further analysis: compound should reduce cell viability (PI-staining) to ≤ 50% of control and clonogenicity to ≤ 20% of control. Next, the selected compounds were used at lower concentrations for dose-response curves. The most potent compounds were then investigated for their ability to induce apoptosis, to influence the frequency of ABCB5-positive cells and label-retaining slow-cycling cells, and to alter gene expression.

The Natural Products Set II, consisting of 120 compounds (Table S1 in File SI) derived from the DTP Open Repository collection of 140,000 compounds, was screened to identify compounds effective against melanoma cells. In the pre-screen of compounds, two cell lines derived from advanced melanoma were used, one from nodular melanoma (DMBC12) [15] and one from superficial spreading melanoma (DMBC11) [37]. Both populations were grown in SCM in an anchorage-independent manner as multicellular 3-dimensional spheroids. In the primary screening, melanoma cells from dissociated spheroids were exposed to each compound at a single concentration of 5 µM. Both, non-clonogenic and clonogenic assays were used in parallel (Fig. 1).

### Short-Term Cytotoxic and Cytostatic Effects of Natural Compounds on Melanoma Cells

**Figure 2:**
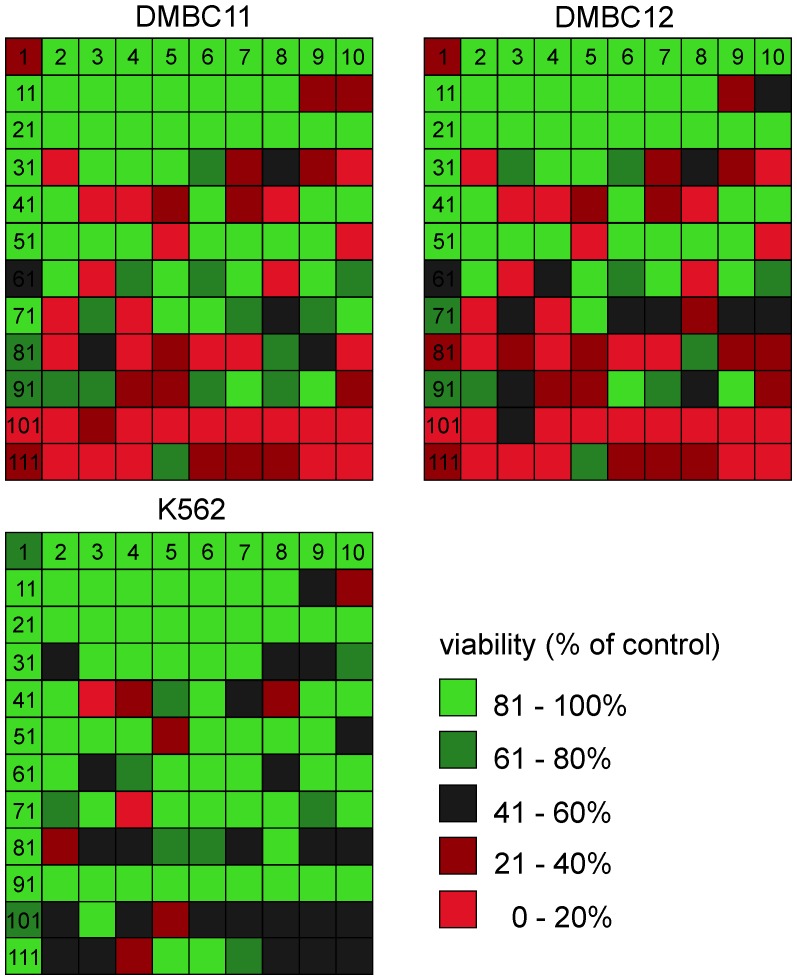
Viability of melanoma cells was substantially reduced by several natural compounds used at 5 µM. Viability was measured after 45% of vehicle (0.05% DMSO)-treated control. For comparison, the leukemia cell line K562 was used. Each square represents the response of melanoma or leukemia cells to one out of 120 compounds. Colors indicate the level of cell response. For clarity the numbers designated in the present study to the tested compounds (Table S1 in File SI) are put in the first raw and the first column. See Figure S2 in File SI for quantitative data.

**Figure 3:**
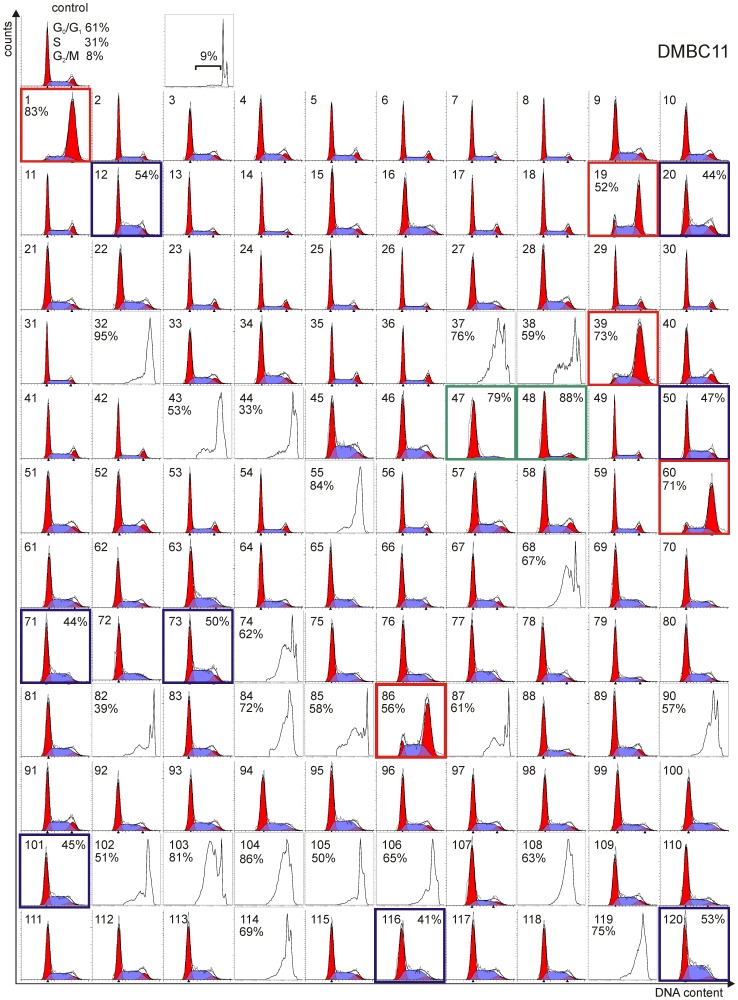
Several natural compounds at 5 µM caused accumulation of melanoma cells in subG_1_ and only few compounds induced the cell cycle arrest. Representative histograms of DMBC11 cells treated with natural compounds for 30_1_ did not exceed 40%, histograms were analyzed using ModFit software to calculate the percentages of cells in each cell cycle phase. Histograms showing cell cycle arrest in G_0_/G_1_ phase were marked with green frame, in S phase with blue frame and G_2_/M in red frame, and the percentages of melanoma cells arrested in those phases are indicated. When accumulation of melanoma cells in subG_1_ exceeded 40%, FACSuit software was used and the percentages of dead cells are indicated. Results obtained for DMBC12 cells are shown in Figure S3A in File SI. Effects of lower concentrations for the most cytotoxic compounds or of longer exposure for compounds ineffective at 30 h are included in Figure S3B in File SI.

Four non-clonogenic methods were chosen for the initial screening. Changes in the viable cell numbers were determined based on the quantification of cytosolic acid phosphatase activity (APA assay) (Fig. S1A in File SI) and by flow cytometry using an automated cell viability analyzer (volumetric assay) (Fig. S1B in File SI). Cytoxicity of the compounds was measured by flow cytometry after PI staining, either as the frequency of PI-positive cells (Fig. 2 and Fig. S2 in File SI) or as percentage of cells in subG_1_ fraction. Histograms for those compounds that accumulated more than 40% of melanoma cells in subG_1_ fraction are included in Fig. 3 and Fig. S3A in File SI. All these assays were used to assess drug activity after short incubation, either after 45 h for the quantification of fractions of viable and PI-positive cells, or after 30 h for percentages of cells in subG_1_ fractions. In parallel, natural compounds at 5 µM were assessed in drug-resistant, p53-deficient leukemic cell line K562 (Fig. 2, Fig. S1-S2 in File SI). The comparison of cytotoxicity of natural products against melanoma and leukemia cells (Fig. 2) revealed that several compounds active against melanoma cells were not active in K562 cells. For example, streptonigrin (32), crassin (68) and geldanamycin analog (72) reduced viability of melanoma cells to less than 3% of control, but the reduction of viability of K562 cells did not reach 50% of control. Melanoma cells were also much more sensitive to fastigilin B (63), bactobolin (84), didemnin B (104), siomycin A (107), toyocamycin (108), geldanamycin (110) and tubulosine (119) among others (Fig. 2). K562 cells, in turn, were much more sensitive to lapachone (20) than melanoma cells.

In cell cycle analysis, several compounds at the concentration of 5 µM accumulated melanoma cells either in different cell cycle phases or generated damaged cells collected in subG_1_ (Fig. 3 for DMBC11 cells and Fig. S3A in File SI for DMBC12 cells). Colchicine (1), rotenone (19), vincristine (39), maytansine (60) and rhizoxin (86) arrested the majority of melanoma cells in G_2_/M phase. Resorufin (12), michellamine B (101) and fumagillin (120), among others arrested cells in S phase, whereas brefeldin A (47) and camptothecin (48) accumulated cells in G_0_/G_1_ or subG_1_ depending on the drug and cell line. Several compounds, which at 5 µM caused no effects on cell cycle after treatment for 30 h, were used in the subsequent experiment for 45 h, whereas compounds causing accumulation of cells in subG_1_ were tested at 1 µM, and in case of the most potent compounds at 0.1 µM (Fig. S3B in File SI). Streptonigrin (32) generating a large population of cells in subG_1_ at 5 µM, accumulated DMBC12 cells in S phase when used at 0.1 µM.

### Pre-screen of Clonogenic Capacity as a Long-Term Effect of Natural Compounds on Melanoma Cells

**Figure 4:**
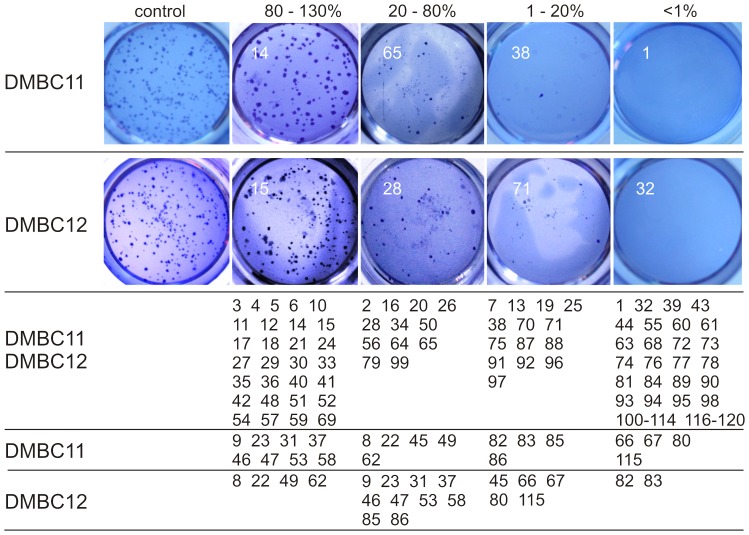
Several natural compounds at 5 µM influenced the clonogenic growth of melanoma cells. Cells were incubated in compound-containing medium for 4 h and then they were grown on soft agar for 14 days in the presence of drug-free medium. Cell colonies were stained with crystal violet and counted. Compounds were grouped based on their anti-clonogenic activity expressed as percentage of control treated with vehicle (0.05% DMSO). At least two independent experiments were performed in duplicates. See Figure S4 in File SI for quantitative data.

A clonogenic assay was employed to investigate effects of natural compounds on self-renewing capacity of melanoma cells. Cells were incubated with the compounds only for 4 h, and long-term effects of this incubation were assessed as capability of forming spheres in soft agar after 2 weeks. Forty eight and forty six compounds at 5 µM reduced the numbers of clones formed in agar to ≤1% of control in DMBC11 and DMBC12 populations, respectively (Fig. 4 and Fig. S4 in File SI). As clonogenic cells might divide more slowly, and the colony might not reach the appropriate size within 2 weeks, for several compounds the colony counting was done one week later. There were no substantial discrepancies between the number of clones obtained after two and three weeks (data not shown).

### Comparison of Short- and Long-Term Effects of Active Natural Compounds on Melanoma Cells

**Figure 5:**
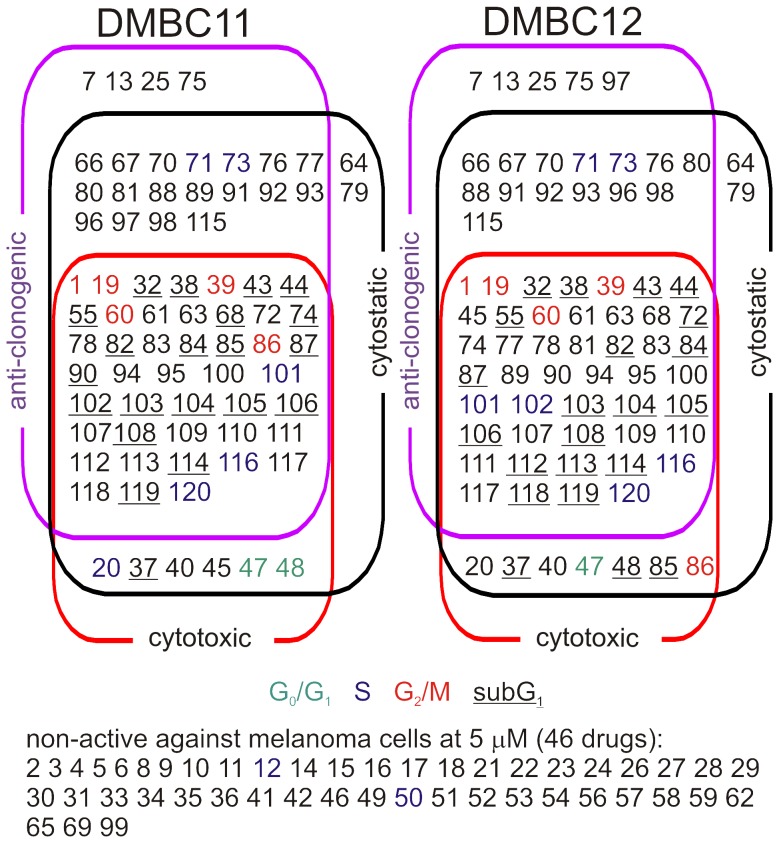
The summary of natural compound activities at 5 µM. After initial screening, compounds were grouped based on their activities. A compound was defined as anti-clonogenic when it reduced the percentage of clones formed in soft agar to less than 20% of control treated with vehicle (0.05% DMSO). A compound was named cytostatic when it reduced the viable cell number to less than 50% of control using viable cell number counting, and cytotoxic using the flow cytometry after PI-staining. Numbers corresponding to compounds that accumulated melanoma cells in subG_1_ are underlined. Compounds that caused cell cycle arrest are marked in green for G_0_/G_1_ phase, blue for S phase and red for G_2_/M phase. Several compounds (46) were not active in any assay. Few compounds were cytotoxic but not markedly influenced the numbers of colonies formed in agar. Few other compounds exerted their effects only on clonogenic cells or reduced clonogenicity below 20% of control, caused cytostatic/cytostatic effect without inducing substantial cell death. Several compounds were cytostatic and cytotoxic and in addition they efficiently eradicated cells with clonogenic potential. They were in the focus of the further study. See Table S1 in File SI for the names of the compounds corresponding to numbers shown in the Figure.

After the initial screening performed at 5 µM, each compound from The Natural Products Set II could be assigned to one of five categories (Fig. 5). Forty six compounds at 5 µM were not active in any of employed assays in both tested populations. They were excluded from further analyses. Some of those non-active compounds such as curcumine (27) or rapamycin (69) are well-characterized for their anticancer activity but apparently at 5 µM they were not active against anchorage-independent melanoma cells. Several compounds were capable of inducing cell death within the first two days, and in addition they efficiently eradicated cells with clonogenic potential. Those compounds were considered as very potent, and their activities, both non-clonogenic and clonogenic, were measured at lower concentrations in the following experiments. Two drugs, actinomycin D (105) and cyclophosphamide (117), were excluded from further analysis, as they are very well-characterized for their *in vitro* and *in vivo* anticancer activity. In addition, parthenolide (61) was also excluded as our detailed report describing its effects on anchorage-independent melanoma cells, was published recently [36]. As priority was given to compounds that reduced the number of melanoma cells showing self-renewing capacity, selected compounds from the group of anti-clonogenic but not highly cytotoxic were also further analyzed.

First, the cytotoxic effects of potent natural compounds were confirmed in six patient-derived cell lines, including four additional cell lines derived from surgical specimens of nodular melanoma, DMBC2, DMBC8, DMBC10 [15] and DMBC9 [36]. Concentrations of the compounds were lowered to 1 µM and 0.1 µM, and in some cases to 0.01 µM or even 0.001 µM, until IC_50_ effect was reached (Table S2 in File SI). In general, only small differences between responses of melanoma cells from different DMBC populations were visible at drug concentrations of 1 µM and 0.1 µM. DMBC8 cells seemed to be less sensitive to several compounds than other populations.

**Figure 6:**
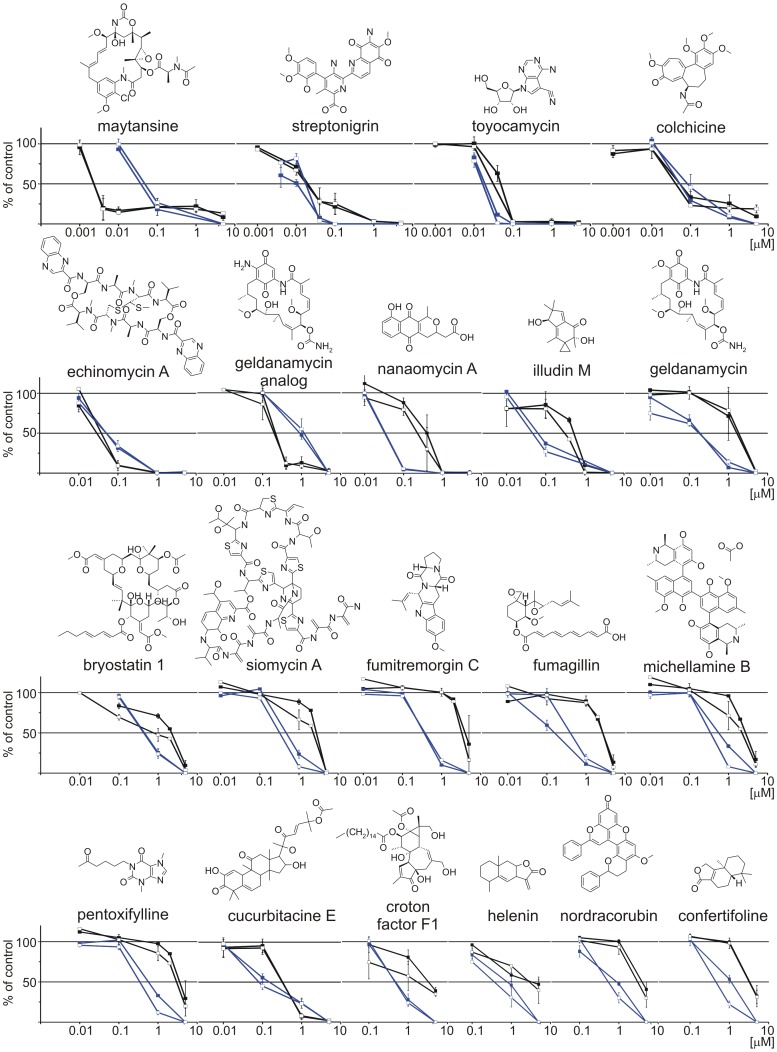
Several compounds were more anti-clonogenic than cytotoxic whereas others exerted opposite activities. Dose-response curves were prepared for compounds showing high anti-clonogenic and/or cytotoxic potential. Blue curves, anti-clonogenic activity; black curves, cytotoxic activity. The graphs summarized the results of at least 3 independent experiments performed in triplicates using DMBC11 (filled square) and DMBC12 (open square) cell lines. Chemical formulas of tested compounds are included. Dose-response curves for less potent compounds are shown in Figure S5 in File SI.

Next, dose–response curves were prepared for 45 compounds exerting strong anti-clonogenic and/or cytotoxic potentials (Fig. 6 and Fig. S5 in File SI). Based on the dose-response curves, the ranking list of the most potent compounds from the Natural Products Set II was prepared (Table 1). Anti-clonogenic activity was ranked above cytotoxic activity. IC_50_ values of seven compounds were below 0.1 µM when the drug influence on the capacity of forming clones in soft agar was evaluated. Among those compounds, maytansine (60) exerted a stronger overall cytotoxic effect than anti-clonogenic effect, streptonigrin (32), toyocamycin (108), colchicine (1) and echinomycin A (102) had similar potency in both activities, whereas nanaomycin A (74) and illudin M (114) were more potent in eradicating cells with the clonogenic potential than in reducing viability within the short-term incubation. Similar phenomena were observed for several compounds that were effective at higher concentrations. For instance, the IC_50_ values for anti-clonogenic compounds, bryostatin 1 (112), siomycin A (107), fumitremorgin C (118), fumagallin (120) michellamine B (101) and pentoxifylline (100) were in the range 0.1 – 1 µM in the clonogenic assay but in the range 1 – 5 µM in the viability assay.

**Table 1:** Comparison of cytotoxic and anti-clonogenic activities of highly potent natural compounds from Natural Products Set II.

A. ‘anti-clonogenic drugs’: more potent against clonogenic cells than fast-cycling				
drug			IC_50_ in the range of concentrations [μM]	
NCI number	this study number	name	for anti-clonogenic activity	for cytotoxic activity
267461	74	nanaomycin A	**0.01 – 0.1**	0.1 – 1
400978	114	illudin M	**0.01 – 0.1**	0.1 – 1
122750	110	geldanamycin	**0.1 – 1**	1 – 5
339555	112	bryostatin 1	**0.1 – 1**	1 – 5
285116	107	siomycin A	**0.1 – 1**	1 – 5
719655	118	fumitremorgin C	**0.1 – 1**	1 – 5
9168	120	fumagillin	**0.1 – 1**	1 – 5
661755	101	michellamine B	**0.1 – 1**	1 – 5
637086	100	pentoxifylline	**0.1 – 1**	1 – 5
338250	89	croton Factor F1	**0.1 – 1**	1 – 5
302289	81	helenin	**0.1 – 1**	1 – 5
376248	95	nordracorubin	**0.1 – 1**	1 – 5
375294	94	confertifoline	**0.1 – 1**	1 – 5
221019	111	wortmannin	**0.1 – 1**	1 – 5
349438	113	4-ipomeanol	**0.1 – 1**	1 – 5
210236	68	crassin	**0.1 – 1**	1 – 5
614552	116	castanospermine	**0.1 – 1**	1 – 5
307981	83	lonchocarpic acid	**0.1 – 1**	1 – 5
401005	115	pleurotin	**0.1 – 1**	> 5

B. ‘cytotoxic drugs’: equally or more potent against fast-cycling cells than against clonogenic cells				
153858	60	maytansine	0.01 – 0.1	**0.001 – 0.01**
45383	32	streptonigrin	0.01 – 0.1	0.01 – 0.1
63701	108	toyocamycin	0.01 – 0.1	0.01 – 0.1
757	1	colchicine	0.01 – 0.1	0.01 – 0.1
526417	102	echinomycin A	0.01 – 0.1	0.01 – 0.1
106399	109	cucurbitacin E	0.1 – 1	0.1 – 1
325319	104	didemnin B	0.1 – 1	0.1 – 1
131547	119	tubulosine	0.1 – 1	0.1 – 1
333856	106	tetrocarcin A	0.1 – 1	0.1 – 1
67574	39	vincristine	0.1 – 1	0.1 – 1
325014	84	bactobolin	0.1 – 1	0.1 – 1
85236	44	helenalin	0.1 – 1	0.1 – 1
305222	82	cytochalasin H	0.1 – 1	0.1 – 1
82151	43	daunorubicin	0.1 – 1	0.1 – 1
332876	87	hispanolone	0.1 – 1	1 – 5
255109	72	geldanamycin analog	∼ 1	0.1 – 1
332598	86	rhizoxin	1 – 5	**0.1 – 1**
330753	85	baccatin III	1 – 5	1 – 5
62709	38	imidazoquinoline	1 – 5	1 – 5
26258	19	rotenone	1 – 5	1 – 5
345647	90	chaetochromin	1 – 5	1 – 5
176503	63	fastigilin B	1 – 5	1 – 5
287088	78	physalin B	1 – 5	1 – 5
85239	45	parthenin	1 – 5	1 – 5
122819	55	teniposide	1 – 5	1 – 5
122023	103	valinomycin	1 – 5	1 – 5, >5

### Induction of Apoptosis in Melanoma Cells Exposed to Selected Compounds

**Figure 7:**
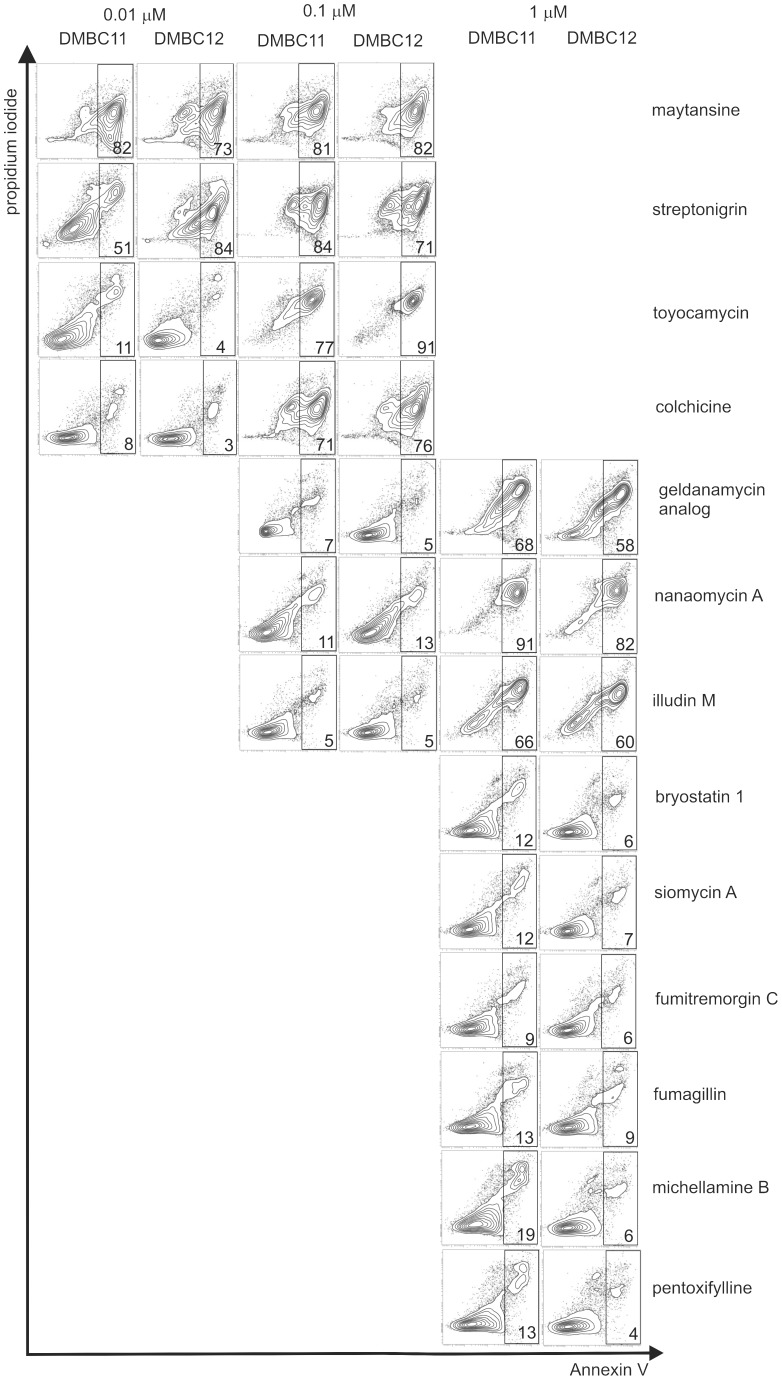
Anti-clonogenic compounds did not induce apoptosis in melanoma cells. Induction of apoptosis was determined by flow cytometry after Annexin V/propidium iodide staining. Typical contour plots of melanoma cells treated with compounds at indicated concentrations are shown. Numbers in the rectangles indicate the mean percentages of all Annexin V-positive cells, both PI negative (early apoptosis) and PI positive (late apoptosis/necrosis) (n = 2). Several compounds e.g., nanaomycin A or pentoxifylline, used at the concentrations that were effective against clonogenic cells, did not induce apoptosis. The percentages of Annexin V-positive cells in vehicle-treated cells did not exceed 6% (not shown).

Flow cytometric analysis of Annexin V/PI-stained cells revealed that highly cytotoxic compounds induced apoptosis in melanoma cells, whereas compounds eradicating mainly cells with the capacity to form clones (Table 1) did not trigger apoptosis at concentrations effective for their anti-clonogenic activity (Fig. 7). For example, maytansine (60) induced apoptosis in the majority of cells at the concentration as low as 0.01 µM. On the contrary, nanaomycin A (74), which at 0.1 µM almost completely eradicated cells with clonogenic potential did not induce apoptosis at this concentration.

### Influence of Selected Compounds on Cell Division of Fast- and Slow-Cycling Subpopulations

**Figure 8:**
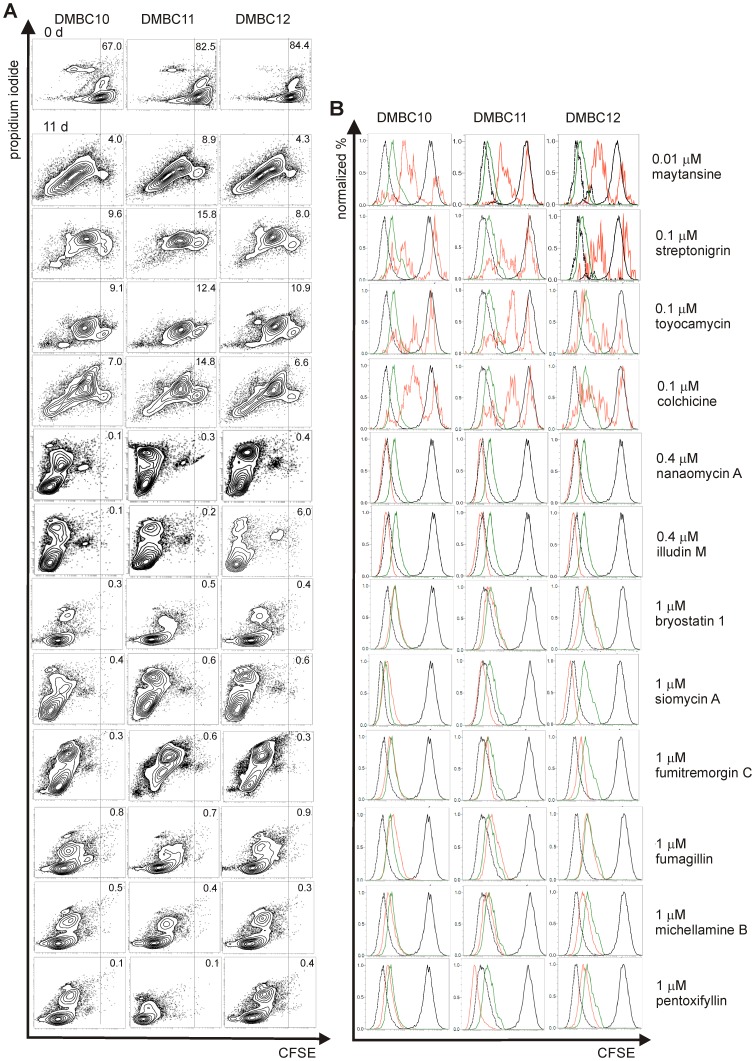
Highly cytotoxic compounds were not active against a subpopulation of slow-cycling label-retaining melanoma cells. Melanoma cells were stained with CFSE and treated with vehicle (control) or selected compounds, and the remaining CFSE fluorescence was measured by flow cytometry. (A) Percentages of viable, non-divided cells in populations DMBC10, DMBC11 and DMBC12 treated with compounds or vehicle (control) are shown on representative contour plots. (B) CFSE fluorescence intensity is shown as green lines for control cultures (day 11^th^), red lines for drug-treated cultures (day 11^th^), solid black lines for cells before the start of treatment (day 0) and dashed black lines for unstained control cells. For most of the compounds, experiment was performed only once, however, results were consistent between all three cell lines.

We further characterized the top-ranked anti-clonogenic and/or cytotoxic compounds (Table 1) for their impact on cell division. To analyze their effects on subpopulations of fast-dividing and slow-cycling cells, the CFSE assay was employed. CFSE is a prodrug for an intracellular vital green-fluorescent dye that is partitioned evenly between daughter cells upon cell division. It had no effect on cell viability and proliferation rates (data not shown). Over the course of eleven days, five days of drug treatment followed by six days of recovery period, dividing cells diluted out CFSE and decreased in fluorescence intensity (Fig. 8). At 11^th^ day, however, in cultures of melanoma cells treated with maytansine (60) at 0.01 µM and streptonigrin (32), toyocamycin (108) and colchicine (1) at 0.1 µM, a small fraction of viable, label-retaining cells (LRCs) was still present indicating that a distinct subpopulation of slow-dividing cells remained unaffected by these compounds (Fig. 8). The fractions of LRCs were also observed when maytansine at higher concentration of 0.1 µM and streptonigrin at lower concentration of 0.01 µM were used (data not shown). On the contrary, no LRCs were left in the populations treated with compounds such as nanaomycin A (74), illudin M (114), bryostatin 1 (112), siomycin A (107), fumitremorgin C (118), fumagillin (120), michellamine B (101) and pentoxifylline (100) (Fig. 8) at the concentrations not substantially affecting overall cell viability, but sufficient to eliminate most cells with clonogenic potential (Table 1).

### Influence of Natural Compounds on Stemness-Associated Molecules and Pathways

**Figure 9:**
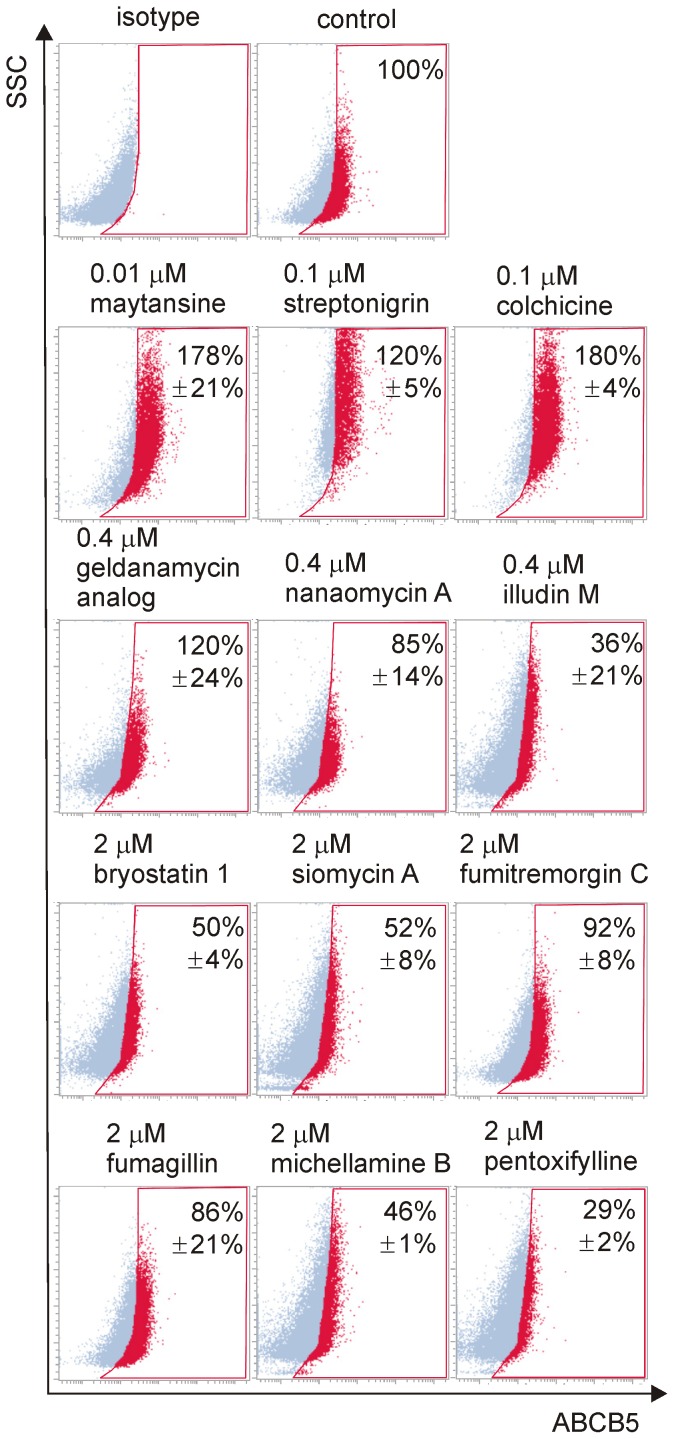
Selected natural compounds exerted diverse effects on cells expressing ABCB5 transporter. Representative dot plots showing ABCB5-positive cells in DMBC12 cell line treated with vehicle and selected compounds. The frequency of ABCB5-positive cells in compound-treated population are expressed as the percentages of control treated with vehicle. Treatment with maytansine and colchicine increased the frequency of cells expressing ABCB5 transporter, whereas illudin M, bryostatin 1, siomycin A, fumagillin, michellamine B and pentoxifylline reduced the frequency of those cells. Data are the mean ± SD of two independent experiments.

**Figure 10:**
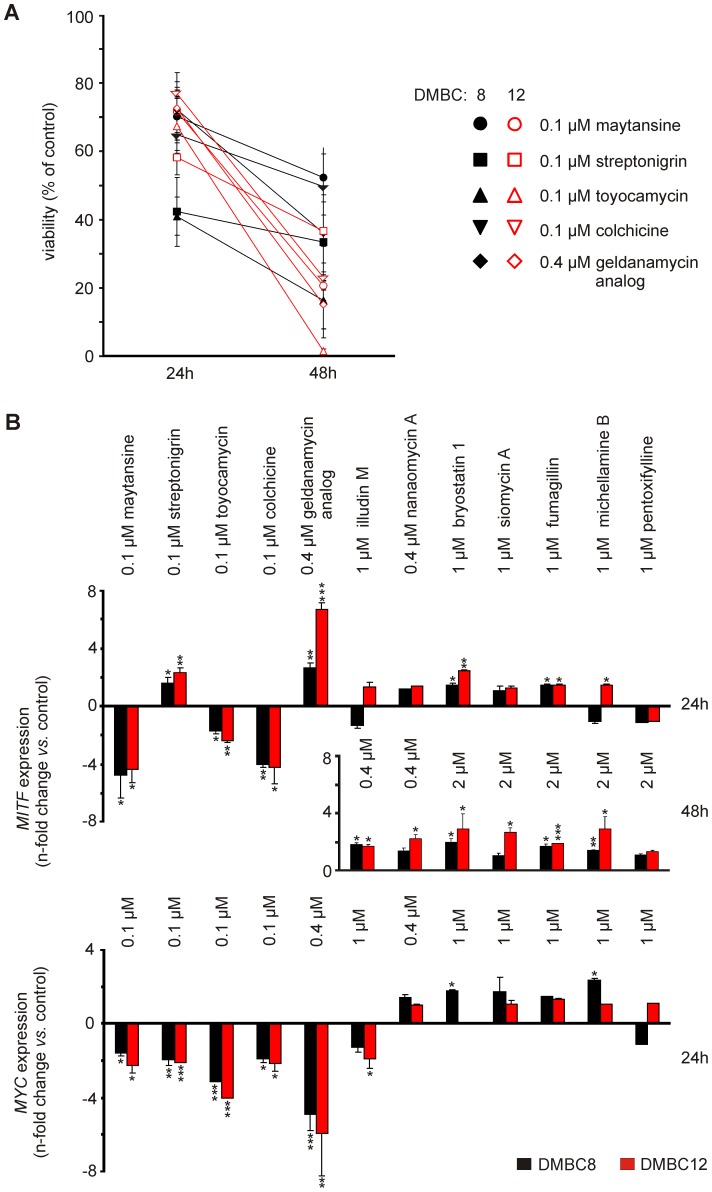
Selected natural compounds induced diverse effects on the expression of *MITF* and *c-MYC*. (A) Cell viability was measured for highly cytotoxic compounds to choose a concentration not reducing cell viability below 40% of control after 24 h of treatment. (B) qRT-PCR was used to assess the fold change in the expression of *MITF* and *c-MYC* after treatment with selected compounds at indicated concentrations and time of exposure. (*P<0.05; **P<0.01; ***P<0.001).

Drug resistance is considered as an element of cancer stem cell characteristics, and the ABCB5 transporter closely associated with melanoma-initiating cell phenotype is responsible for the efflux of multiple chemotherapeutic agents [2], [22], [23]. Flow cytometry was used, and the representative dot plots obtained after 45 h exposure of DMBC12 cells to selected compounds are shown in Fig. 9. Maytansine (60) at 0.01 µM and colchicine (1) at 0.1 µM increased the proportion of ABCB5-positive cells, whereas 0.4 µM illudin M (114) and 2 µM bryostatin 1 (112), siomycin A (107), fumagillin (120), michellamine B (101) and pentoxifylline (100) preferentially killed those cells. Pentoxifylline (100) was the most efficient in reducing the proportion of ABCB5-positive cells. The drug influence on the expression of two targets of Wnt/β-catenin signaling, *MITF* and *c-MYC*, was analyzed by qRT-PCR. Two primary melanoma cultures, DMBC8 and DMBC12, showing the highest difference in *MITF* expression were chosen. For investigations of the influence of drug on gene expression, concentrations resulting in viability above 40% of control were chosen (Fig. 10A). *MITF* expression was significantly inhibited by 0.1 µM maytansine (60), toyocamycin (108) and colchicine (1), whereas its expression was significantly higher in melanoma cells treated with 0.1 µM streptonigrin (32) and 0.4 µM geldanamycin analog (72) for 24 h (Fig. 10B). This tendency was well preserved when cells were treated for 48 h with these compounds at lower concentrations (not shown), despite the marked influence on cell viability. When compounds exerting low effects on *MITF* expression after 24 h were tested at higher concentrations (2 µM) for 48 h, a significant increase in *MITF* expression was observed for most of those compounds. *MITF* expression was more enhanced in DMBC12 than in DMBC8 cells after treatment with the majority of compounds. This may be due to the difference in the basal level of *MITF* expression between DMBC8 and DMBC12 cells; DMBC8 cells had 18-fold higher basal level of the MITF transcript than DMBC12 cells (data not shown). *c-MYC* expression was significantly reduced after 24 h of treatment mainly by compounds markedly affecting proliferation. No substantial changes were induced in *SOX2* expression (data not shown).

## Discussion

The present study re-evaluated compounds from The Natural Products Set II with a view to determining their potential against melanoma cells grown as anchorage-independent spheroids. These 3-dimentional cultures exhibit a more heterogeneous phenotype than monolayers maintained in the presence of serum [16], [18]. The idea was to select compounds most potent in affecting the self-renewing capacity in the populations with the high enough content of melanoma cells exerting this property [15]. Those compounds might be overlooked when more uniform populations such as monolayers are used. Those compounds might be also disregarded in the selection process when assays measuring exclusively cytotoxic or cytostatic short-term effects are employed. In our study a broad spectrum of screening methods was used to rank 120 natural compounds not only according to their cytotoxic effects but mainly according to their potential to eradicate melanoma stem-like cells. Compounds that were selected as highly anti-clonogenic and/or cytotoxic were further investigated for their potential to affect slow-cycling cells, to influence the frequency of ABCB5-positive cells and to alter the expression of *MITF* and *c-MYC*.

The present screening re-identified a number of compounds that have already been tested against melanoma cells, and streptonigrin, bryostatin 1, siomycin A and pentoxifylline were among the compounds classified as the top ranked ones in this study. Streptonigrin was previously found to be active against multiple cancer cells [38], however, its clinical usage in cancer treatment is limited by severe and prolonged side effects. Streptonigrin is converted by NAD(P)H:quinine oxidoreductase (NQO1) to the more active hydroquinone form. Therefore, tumors containing high levels of this enzyme might be sensitive to streptonigrin at concentrations that do not generate severe side effects [39]. It was also shown that streptonigrin at 0.05 µM markedly decreased clonogenic survival of pancreatic cancer cells expressing NQO1 at elevated levels but not of colorectal cancer cells with lower levels of this enzyme [40]. Streptonigrin has been shown to inhibit β-catenin/TCF signaling by suppression of TCF-mediated DNA binding and to reduce the level of nuclear β-catenin [41]. Its growth inhibitory properties were observed mainly in β-catenin-activated embryonic cells and cancer cells, and were associated with decreased expression of c-MYC, cyclin D and AXIN2 [41]. Results from the present study support some of those findings. For example, streptonigrin was more cytotoxic against melanoma cells than against leukemia K562 cells which might indicate a cancer type specificity, probably connected with the NQO1 level. As NQO1 is over-expressed in most melanoma cell lines in comparison with normal melanocytes [42], even low concentration of streptonigrin might be sufficient to kill melanoma cells. Secondly, streptonigrin at the concentration as low as 0.04 µM was highly effective against melanoma cells with clonogenic potential which is in line with the previous finding for pancreatic cancer cells [40]. Streptonigrin significantly reduced the expression of *c-MYC*, but it increased the expression of *MITF*, and this interesting result needs to be further investigated. Compounds less cytotoxic than streptonigrin, bryostatin 1, siomycin A and pentoxifylline, were selected based on their activity against melanoma cells exerting high self-renewing capacity. In addition, all of them were more cytotoxic to melanoma cells than to K562 cells. Those compounds substantially decreased the frequency of ABCB5-positive melanoma cells and removed the slow-cycling label-retaining cells at concentrations that did not trigger apoptosis. In a previous study [43], bryostatin 1 increased HLA class II protein levels crucial for the stimulation of CD4+ T cells. It also enhanced the expression of costimulatory molecules (CD80 and CD86) in melanoma cells and induced melanoma cell differentiation. It was implied that this drug could be used as a chemo-immunotherapeutic agent for reducing tumorigenic potential and preventing melanoma recurrence. Our present data suggest that bryostatin 1 is either capable of stimulating melanoma cell differentiation or selectively eradicates more primitive stem-like melanoma cells. The next compound, siomycin A was shown previously to reduce viability in melanoma cell lines [44]. Treatment of stem-like glioblastoma multiforme cells with siomycin A resulted in arrested self-renewal [45]. In the present study, the reduction of cells with clonogenic capacity, ABCB5-positive and slow-cycling cells indicates that siomycin A at 1 µM is more effective against melanoma stem-like cells than against fast-cycling cells. Pentoxifylline, the next compound selected in our study, is clinically used in patients with chronic peripheral arterial disease to increase blood flow and to enhance tissue oxygenation. In melanoma cell lines, pentoxifylline combined with irradiation significantly increased radiotoxicity in a *TP53* mutant cell line, effectively suppressed DNA double-strand break repair [46], inhibited in G_1_-S phase transition [47], caused glutathione depletion and increased glutathione-S-transferase activity [48]. In *in vivo* experiments, it showed anti-metastatic activity [49], and significantly inhibited subcutaneous melanoma xenograft growth and angiogenesis without any toxicity [50], [51]. In the present study, pentoxifylline was more efficient in reducing percentages of cells with clonogenic potential than in affecting overall cell viability. More importantly, pentoxifylline might be also considered as capable of reducing availability of ABCB5 transporter in the cell membrane.

Several other natural compounds not considered previously as anti-melanoma agents were selected in the present study. From this group comprising toyocamycin, nanaomycin A, illudin M, fumitremorgin C, fumagillin and michellamine B, geldanamycin and its analog, two natural agents: toyocamycin and nanaomycin A were capable of eradicating clonogenic melanoma cells at the concentration as low as 0.1 µM. Interestingly, toyocamycin was also highly cytotoxic at this concentration. Nanaomycin A was less cytotoxic, and at 0.1 µM it reduced the overall viability to about 80% of control and did not increased the percentages of Annexin V-positive cells. Toyocamycin blocks RNA synthesis and ribosome function [52], [53]. Most recently, it has been shown that it binds to the ATP-binding site of Rio1 kinase, an enzyme involved in the maturation of 40S RNA [54]. Toyocamycin was also recognized as a potent inhibitor of ER stress-induced XBP1 mRNA splicing [55]. At nanomolar concentrations, it induced apoptosis of multiple myeloma cells including bortezomib-resistant cells and inhibited growth of xenografts [55]. The clinical application of toyocamycin is limited because of its toxicity. There have not yet been any *in vivo* preclinical studies or clinical trials with nanaomycin A, and the number of *in vitro* studies is limited. Nanaomycin A was found to selectively inhibit DNA (cytosine-5-)-methyltransferase (DNMT3B) and reactivate silenced tumor suppressor genes in human cancer cells [56], [57]. Most recently, it was reported that hepatocellular carcinoma side population cells enriched with cancer stem-like cells, possessed a differential DNA methylation status as compared with non-side population cells suggesting that DNA methylation might play a significant role in maintaining the CSC-like status [58]. As our study strongly suggests that nanaomycin A preferentially eradicates cells with self-renewing capacity, it would be interesting to find out whether this drug could eliminate melanoma cells with stem cells characteristics *via* epigenetic regulation. Illudin M exerts anti-proliferative and pro-apoptotic activity by alkylating DNA, RNA and proteins. It has a poor therapeutic index [59], which was improved by structural modification of this compound with ferrocene [60]. The new derivative showed increased apoptotic potential. The esters of illudin M with demethylcantharidinic acid (endothall) and 2,2′-bipyridyl dicarboxylic acid retained the cytotoxicity of the parent compound while displaying significantly improved specificity for tumor cells over normal fibroblasts [61]. In the present study, illudin M at 1 µM markedly reduced cell viability and the frequency of ABCB5-positive cells. At 0.1 µM, illudin M reduced clonogenicity below 40% of control, whereas cell viability decreased to about 80% of control indicating that this compound preferentially eradicated cells with self-renewing capacity. This suggests that illudin M might be further studied *in vivo* at subtoxic concentrations. Geldanamycin and its analog (17-aminogeldanamycin) exert various cellular effects including destabilization of Hsp90 target proteins [62]. In the present study, both compounds were more efficient in reducing viability of melanoma cells than of leukemia cells. Interestingly, the geldanamycin analog showed higher cytotoxic activity than original geldanamycin, however, it was much less potent in eradicating melanoma cells with self-renewing capacity. The geldanamycin analog induced the expression of *MITF*, which is in agreement with a recently published report showing that it caused an increase in MITF protein level [63]. Interestingly, it has been demonstrated that agents increasing *MITF* expression can be exploited in anti-melanoma therapeutic strategies [64]. The next natural drug candidate, fumitremorgin C at micromolar concentrations inhibited expression of the breast cancer resistant protein (BCRP/ABCG2) that mediates transport of chemotherapeutic agents [65]. In the present study, however, fumitremorgin C did not alter the frequency of cells expressing another transporter, ABCB5. It was also shown previously that fumitremorgin C blocked the appearance of side population (SP) phenotype in non-small cell lung cancer cultures [66]. Our study confirmed this finding in melanoma as 1 µM fumitremorgin C very efficiently eradicated primitive cells, which was clearly visible in clonogenic and label retention assays. Fumagillin has been shown to inhibit angiogenesis [67], [68] and irreversibly block methionine aminopeptidase-2 (MetAP-2) through covalent modification [69]. In addition, it significantly reduced cholangiocarcinoma cell proliferation in a dose-dependent manner and the degree of growth inhibition was dependent on the amount of cellular MetAP2 [70]. In the present study, fumagillin at 1 µM was more efficient in eradicating clonogenic cells than in reducing overall cell viability. Michellamine B, known for its anti-yeast and anti-HIV activity, was not yet recognized as having anticancer potential. The present study revealed that it is not highly cytotoxic but markedly reduced the frequency of slow-cycling melanoma cells and ABCB5-positive melanoma cells.

The impact on ABCB5-positive melanoma cells was quite different for two other drugs, maytansine and colchicine. Both compounds were highly cytotoxic but they substantially increased the frequency of ABCB5-positive cells suggesting that these two compounds might be efficiently effluxed from cells expressing this transporter. Maytansine has been tested in several clinical trials involving different tumors but the results of these trials were discouraging due to dose-limiting toxicity and lack of efficacy [71], [72]. Studies performed on NCI's panel of 60 cancer cell lines revealed that maytansine inhibits the assembly of microtubules by binding to tubulin and has the same binding site as rhizoxin [73]. In the present study, maytansine was highly cytotoxic and cell viability was below 22% of control after 48 h treatment in a broad range of concentrations from 0.004 µM to 5 µM. However, when cells were exposed to 0.1 µM maytansine for 24 h, viability was reduced only to 70% of control. This suggests strong time-dependent effects of this compound. Indeed, after the first 30 h of treatment with maytansine at 1 µM or even at 5 µM, the majority of cells were not collected in subG_1_ but in G_2_/M phase, but 18 h later more than 70% of cells were Annexin V-positive at the concentration as low as 0.01 µM. At this concentration, however, maytansine had already lost its anti-clonogenic potential. Probably, the short exposure to the drug (4 h) used in clonogenic assay was not sufficient to obtained long-term effects on cells exerting self-renewing capacity. More interestingly, maytansine did not affect slow-cycling label-retaining cells. Thus, the length of time required to induce anticancer effects and the lack of ability to eradicate all types of melanoma cells, especially those considered as melanoma stem-like cells, could partially explain why maytansine failed in clinical trials. More recently, phase II trials of Her2- and CD19-based antibody conjugates containing a maytansine derivative (maytansine-DM1) have shown good clinical efficacy in breast and B-cell malignancies, respectively [74], [75]. It would be interesting to evaluate this new derivative against melanoma cells, especially against those cells with cancer stem-like characteristics. The next compound, colchicine is used in the treatment of gout, but it has been used also in the therapy of familial Mediterranean fever, pericarditis, and Behçet's disease. It inhibits microtubule polymerization by binding to tubulin [76]. In the present study, colchicine-induced cell death was preceded by G_2_/M arrest of the cell cycle, however, at 0.1 µM colchicine had a limited influence on both fast- and slow-cycling cells. At this concentration it was also not highly effective against clonogenic cells. It has been shown recently that colchicine exerted anti-proliferative effect in hepatocellular carcinoma cell lines [77], but the significance of this finding is diminished by observation that it also reduced viability in normal liver cells [78]. Given these results, colchicine is probably not a good drug candidate for the treatment of melanoma. Its high capacity to select ABCB5-positive and slow-cycling cells could however be used in *in vitro* experiments to enrich melanoma population with cells potentially exhibiting stem-cell characteristics.

## Conclusions

In the present study, numerous agents across different therapeutic categories and modes of action (Table S3 in File SI) have been selected from The Natural Products Set II as highly potent against melanoma cells. As some of natural compounds were capable of targeting melanoma stem-like cells, a subpopulation of tumor cells responsible for cancer initiation, propagation and maintenance, results of our screen represent possible opportunities to repurpose these drugs for the treatment of patients with melanoma. Moreover, because in the clonogenic assay, melanoma cells were not continuously exposed to tested compounds but only for short periods of time, the probability of exceeding the plasma/serum half-life was reduced and the observed long-term effects might be clinical relevant. If we consider that the phenotypic plasticity may facilitate escape of melanoma cells from anti-cancer therapies [79], selected anti-clonogenic drugs should rather be used in combination with highly cytotoxic and/or targeted agents to prevent the regeneration of the heterogeneous pool of cancer cells from a subpopulation that remains unaffected. Nevertheless, our results position bryostatin 1, siomycin A, pentoxifylline, illudin M and michellamine B as drugs against melanoma stem-like cells and potentially resistance-reversing agents capable of reducing ABCB5 expression. Streptonigrin and nanaomycin A, which were even more effective against cells with self-renewing capacity, were not capable of affecting ABCB5-positive cells. It is, however, unclear whether melanoma stem-like cells can be identified through the expression of this transporter [2], [4], [80]. Thus, our results emphasize the need to better understand the role of diverse subpopulations in tumor maintenance and response to therapy.

Another noteworthy aspect of the present study was the demonstration that the most cytotoxic compounds might serve as agents selecting a cancer stem-like cell subpopulation. Maytansine was highly cytotoxic even at 0.01 µM but it did not reduce clonogenicity at this concentration, it selected ABCB5-positive cells and a small subpopulation of slow-cycling cells remained unaffected. This indicates that maytansine and probably also colchicine might be considered as a valuable biochemical tool for future studies of melanoma stem-like cells and certainly warrants further investigation.

## Supporting Information

File S1 **Figure S1. The influence of natural compounds used at a single concentration of 5 µM on viable cell numbers in melanoma (DMBC11 and DMBC12) and leukemia (K562) cell cultures.** Viable cells were assessed by acid phosphatase activity assay (A) or by flow cytometry using an automated cell viability analyzer (B). Data are the mean ± SD of two independent experiments performed in triplicates. **Figure S2. Effects of natural compounds (5 µM) on viability of melanoma cells (DMBC11 and DMBC12) and leukemia cells (K562).** Changes in cell viability were assessed by PI staining and flow cytometry and they are expressed as % of vehicle control. Data are the mean ± SD of two independent experiments performed in triplicates. **Figure S3. The influence of natural compounds on cell distribution in cell cycle and cell death shown as accumulation in subG_1_.** (A) Representative histograms of DMBC12 cells treated with natural compounds at a single concentration of 5 µM for 30 h are shown. When accumulation of melanoma cells in subG_1_ did not exceed 40%, histograms were analyzed using ModFit software to calculate the percentages of cells in each cell cycle phase. Histograms showing cell cycle arrest were marked with green (G_0_/G_1_ phase), blue (S phase) and red (G_2_/M) frames, and percentages of melanoma cells accumulated in each phase are included. When accumulation of melanoma cells in subG_1_ exceeded 40%, FACSuit software was used and the percentages of dead cells are indicated. Results obtained for DMBC11 cells are shown in Figure 3. (B) Effects of lower concentrations for the most cytotoxic compounds or of longer exposure for compounds that were ineffective at 30 h. **Figure S4. The influence of natural compounds used at a single concentration of 5 µM on the clonogenic growth of melanoma cells.** Cells were incubated in drug-containing medium for 4 h and then they were grown on agar for 14 days in drug-free medium. Cell colonies were stained and counted. Anti-clonogenic activity was expressed as percentage of control treated with vehicle. At least two independent experiments were performed in duplicates. **Figure S5. Dose-response curves prepared for compounds exerting anti-clonogenic and/or cytotoxic potentials.** Blue curves, anti-clonogenic activity; black curves, cytotoxic activity. The graphs summarize the results of at least 3 independent experiments performed in triplicates using DMBC11 (filled square) and DMBC12 (open square) cell lines. Chemical formulas of compounds are included. Dose-response curves for more potent compounds are shown in Figure 6. **Table S1. The Natural Products Set II Consisting of 120 compounds.** Table contains names of all compounds and the numbers (underlined) that could be used to get detailed information about compounds from available databases. The upper numbers were given to compounds in the present study, and they appear in brackets in the text and also in some Figures. **Table S2. Viability assessed after 45 h of treatment with selected compounds at indicated concentrations in six different melanoma cell lines derived from surgical specimens.** Cell viability was assessed by flow cytometry after PI staining. Data expressed as % of control treated with vehicle are means ± SD of two independent experiments conducted in triplicates. **Table S3. Activity profiles of natural compounds selected in this study prepared based on a literature search.** Only the main biological activities of compounds are included.
(PDF)
