# Supplementary material for: Natural Compounds' Activity against Cancer Stem-Like or Fast-Cycling Melanoma Cells
Source: PLoS One. 2014 Mar 3;9(3):e90783. doi: 10.1371/journal.pone.0090783 (PMC3940936; doi:10.1371/journal.pone.0090783)
Supplement: File S1 — Figure S1. The influence of natural compounds used at a single concentration of 5 µM on viable cell numbers in melanoma (DMBC11 and DMBC12) and leukemia (K562) cell cultures. Viable cells were assessed by acid phosphatase activity assay (A) or by flow cytometry using an automated cell viability analyzer (B). Data are the mean ± SD of two independent experiments performed in triplicates. Figure S2. Effects of natural compounds (5 µM) on viability of melanoma cells (DMBC11 and DMBC12) and leukemia cells (K562). Changes in cell viability were assessed by PI staining and flow cytometry and they are expressed as % of vehicle control. Data are the mean ± SD of two independent experiments performed in triplicates. Figure S3. The influence of natural compounds on cell distribution in cell cycle and cell death shown as accumulation in subG1. (A) Representative histograms of DMBC12 cells treated with natural compounds at a single concentration of 5 µM for 30 h are shown. When accumulation of melanoma cells in subG1 did not exceed 40%, histograms were analyzed using ModFit software to calculate the percentages of cells in each cell cycle phase. Histograms showing cell cycle arrest were marked with green (G0/G1 phase), blue (S phase) and red (G2/M) frames, and percentages of melanoma cells accumulated in each phase are included. When accumulation of melanoma cells in subG1 exceeded 40%, FACSuit software was used and the percentages of dead cells are indicated. Results obtained for DMBC11 cells are shown in Figure 3. (B) Effects of lower concentrations for the most cytotoxic compounds or of longer exposure for compounds that were ineffective at 30 h. Figure S4. The influence of natural compounds used at a single concentration of 5 µM on the clonogenic growth of melanoma cells. Cells were incubated in drug-containing medium for 4 h and then they were grown on agar for 14 days in drug-free medium. Cell colonies were stained and counted. Anti-clonogenic activity was expressed as pe [file pone.0090783.s001.pdf]

Table S1. The Natural Products Set II consisting of 120 compounds

1<br>ColchIcine<br>757	2<br>Dehydroabi-<br>etic acid<br>2952	3<br>Pomiferin<br>5113	4<br>Chartreusin<br>5159	5<br>Noscapine<br>5366	6<br>Veratridine<br>7524	7<br>Aleuritic<br>acid<br>7668	8<br>Khellin<br>8519	9<br>Fumagillin,<br>alcohol I<br>origin<br>9665	10<br>Argemonine<br>11440
11<br>Aristolochic<br>acid<br>11926	12<br>Resorufin<br>12097	13<br>Aureomycin<br>13252	14<br>Glaucarubine<br>14975	15<br>Amygdalin<br>15780	16<br>Pyrethrosin<br>22070	17<br>Coumestrol<br>22842	18<br>Himbafcine<br>23969	19<br>Cube<br>26258	20<br>Lapachone,<br>beta<br>26326
21<br>Isorescinna-<br>mine,<br>dihydro-<br>29854	22<br>Mangostin<br>30552	23<br>Neohesperi-<br>din<br>31048	24<br>Norlobaric<br>acid,<br>decarboxy-<br>31867	25<br>Bicuculline<br>32192	26<br>Isocorydine<br>32979	27<br>Curcumin<br>32982	28<br>Solanine<br>35611	29<br>Canadine<br>(dl-)<br>36351	30<br>Guercetin,<br>dihydro-<br>36398
31<br>Santonine,<br>ozime<br>42038	32<br>Streptonigrin<br>45383	33<br>Methoxsalen<br>45923	34<br>Carbomycin<br>51001	35<br>Aconitine<br>56464	36<br>Fumagillin<br>dicyclohexyl-<br>amine salt<br>58368	37<br>Tylocrebrine<br>60387	38<br>Streptonigri-<br>naza,<br>izopropylidene<br>62709	39<br>Vincristine<br>sulfate<br>67574	40<br>Ellipticine<br>71795
41<br>Pseudoyo-<br>himbine<br>72116	42<br>Thaspine,<br>acetale<br>76022	43<br>Daunorubicin<br>HCl<br>82151	44<br>Helenalin<br>85236	45<br>Parthenin<br>85239	46<br>Stictic acid<br>87511	47<br>Brefeldin A<br>89671	48<br>Camptothecin<br>94600	49<br>Riboflavin<br>lumichrome<br>96911	50<br>Lagosin<br>105388
51<br>Tirandamycin<br>107067	52<br>Ascochitine<br>114344	53<br>Radicinin<br>118343	54<br>Sporidesmo-<br>lide I<br>122224	55<br>Teniposide<br>122819	56<br>Picrotin<br>129536	57<br>Rifamycin SV<br>133100	58<br>Lankacidin C<br>145118	59<br>Nystatin<br>150817	60<br>Maytansine<br>153858

61 Parthenolide 157035	62 Streptoval C 169627	63 Fastigillin B 176503	64 Antibiotic X-536A 177406	65 Staphylomy-cin S 177858	66 Vermiculine 8140514	67 Antibiotic A-31438 209870	68 Crassin 210236	69 Rapamycin 226080	70 NSC250429 250429
71 NSC250430 250430	72 Geldanamy-cin analog 255109	73 Ehna HCl 263164	74 Nanaomycin 267461	75 Photobarbat-usin I 270914	76 Batyl alkohol 284200	77 Quadrone 284437	78 Physalin B 287088	79 Nigericin 292567	80 Daphnetin-diacetate 301683
81 Bohlmann K2631 302289	82 Cytochalasin H 305222	83 Lonchocarpic acid 307981	84 Bactobolin 325014	85 Baccatin III 330753	86 Rhizoxin 332598	87 Hispanolone 332876	88 Triptolide analog 337783	89 Croton factor F1 338250	90 Chaetochro-min 345647
91 Medicarpin 350085	92 Sequiterpene lactone 326 361902	93 Artemisinine 369397	94 Confertifoline 375294	95 Nordracoru-bin 376248	96 NSC382796 382796	97 Ellagic acid 407286	98 Coynanthine 407306	99 Ergocristine, dihydro- 409663	100 Pentoxifylli-ne 637086
101 Michellamine B 661755	102 Echinomycin 526417	103 Valinomycin 122023	104 Didemnin B 325319	105 Actinomycin D 3053	106 Tetrocarcin A, sodium salt 333856	107 Siomycin A 285116	108 Toyocamycin 63701	109 Cucurbitacin E 106399	110 Geldanamy-cin 122750
111 Wortmannin 221019	112 Bryostatin I 339555	113 4-Ipomeanol 349438	114 Illudin M 400978	115 Pleurotin 401005	116 Castanosper-mine 614552	117 Cyclophosph-amide 26271	118 Fumitremorgin C 719655	119 Tubolosine 131547	120 Fumagillin 9168

The upper numbers represent those ones used in the present study. The lower numbers (underlined) can be used to get detailed information about a drug from available databases.

Figure S1

A. The influence of natural compounds (5 µM) on viable cell numbers in melanoma (DMBC11 and DMBC12) and leukemia (K562) cell cultures assessed by APA assay. Data are the mean ± SD of two independent experimets performed in triplicates.

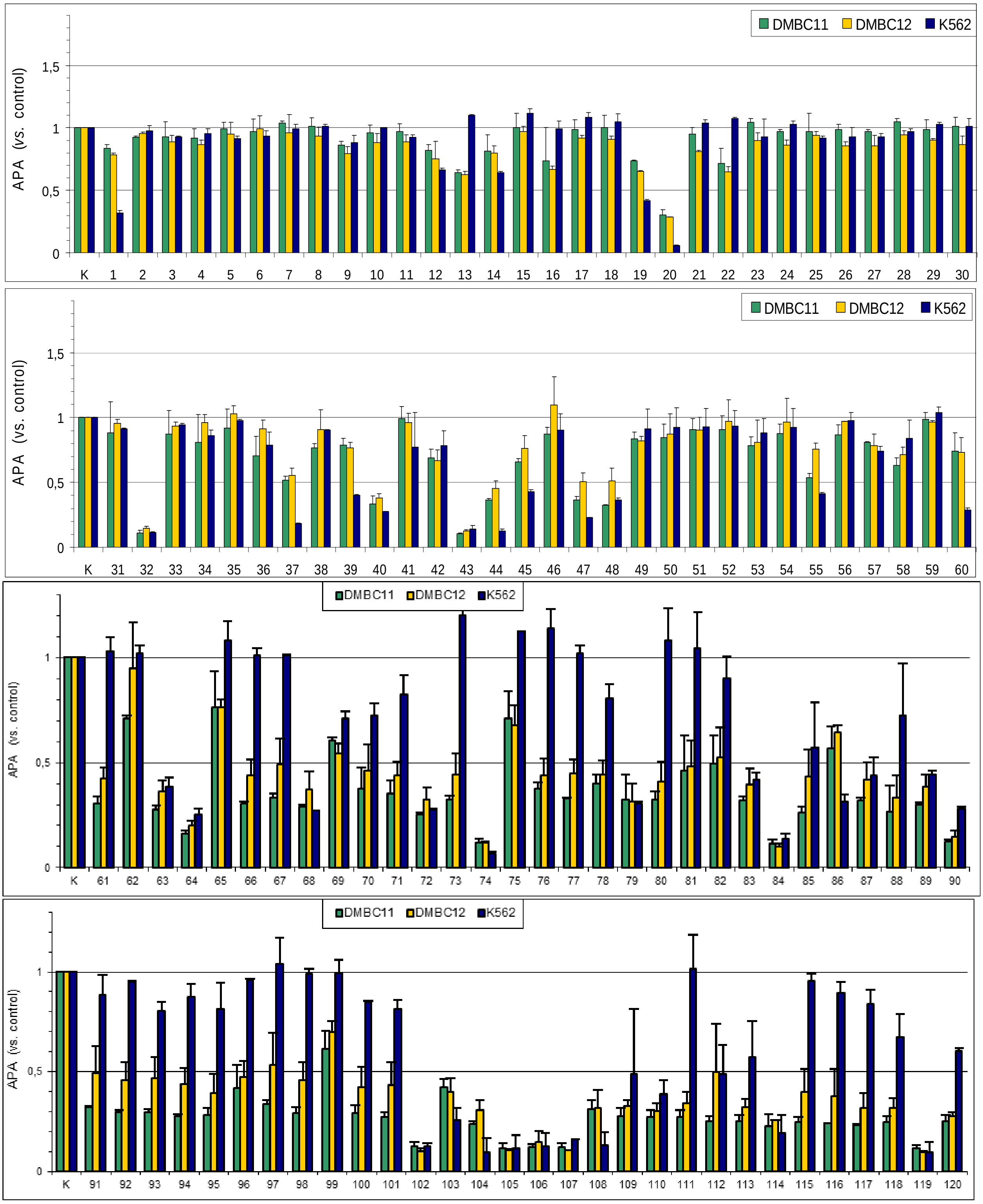

B. The influence of natural compounds (5 μM) on viable cell numbers in melanoma (DMBC11 and DMBC12) and leukemia (K562) cell cultures assessed by flow cytometry using an automated cell viability analyzer (volumetric assay). Data are the mean ± SD of two independent experimets performed in triplicates.

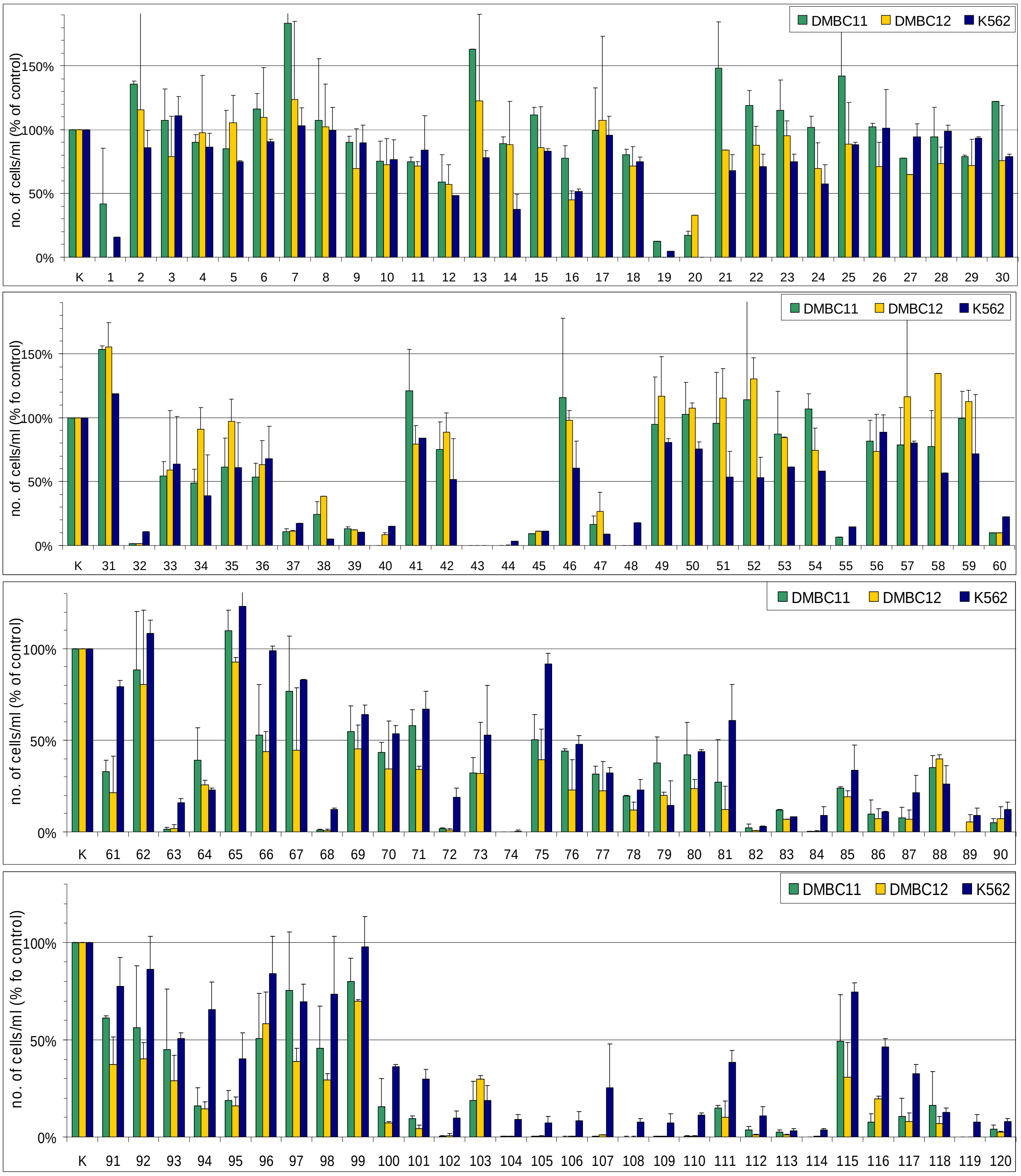

Figure S2

Effects of natural compounds (5 µM) on viability of melanoma cells (DMBC11 and DMBC12) and leukemia cells (K562). Changes in cell viability were assessed by PI staining and flow cytometry and they are expressed as % of vehicle control. Data are the mean ± SD of two independent experiments performed in triplicates.

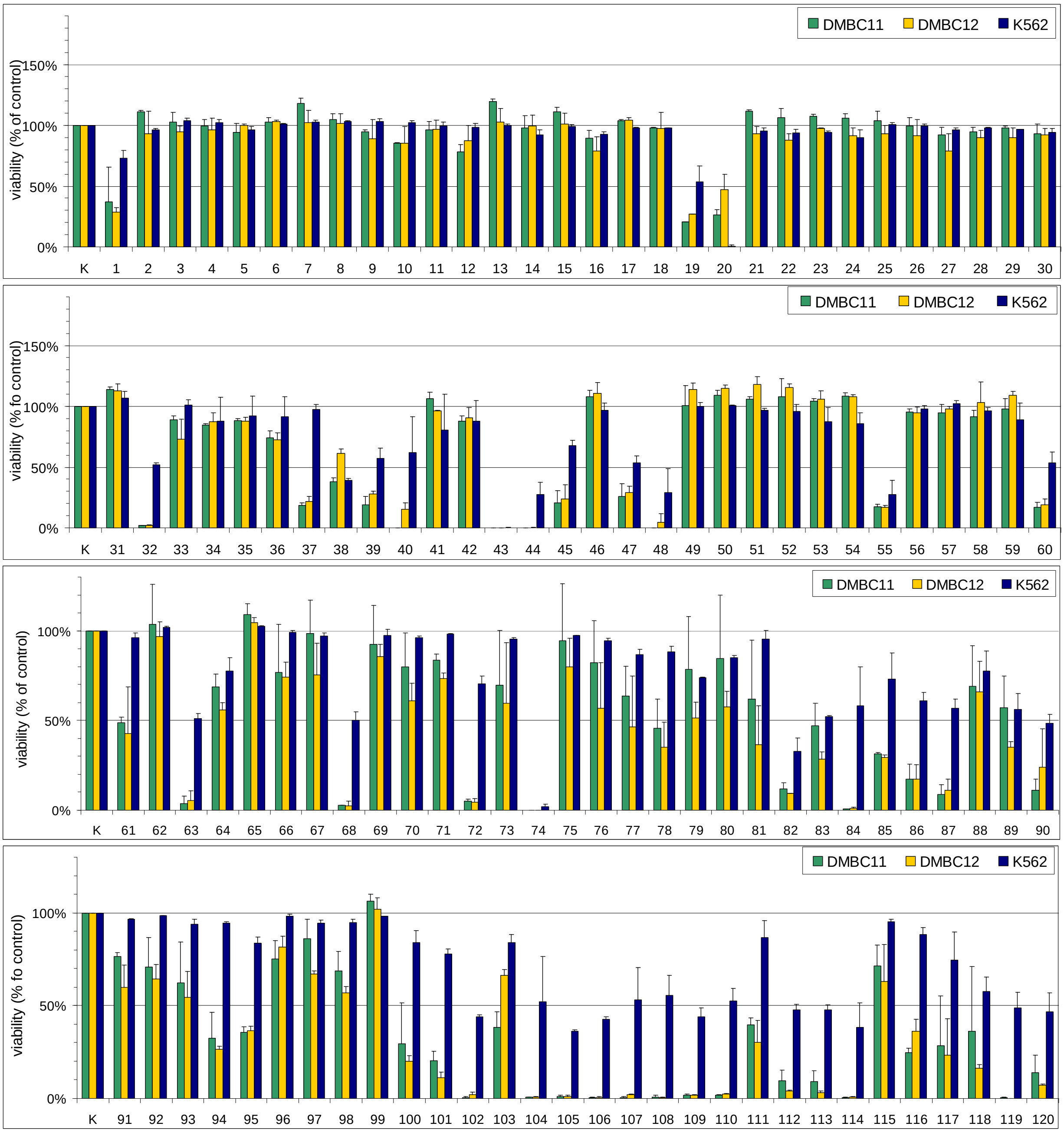

Figure S3A

The influence of natural compounds on cell distribution in cell cycle and cell death shown as accumulation in subG$_1$. (A) Representative histograms of DMBC12 cells treated with natural compounds at a single concentration of 5 µM for 30 h are shown.

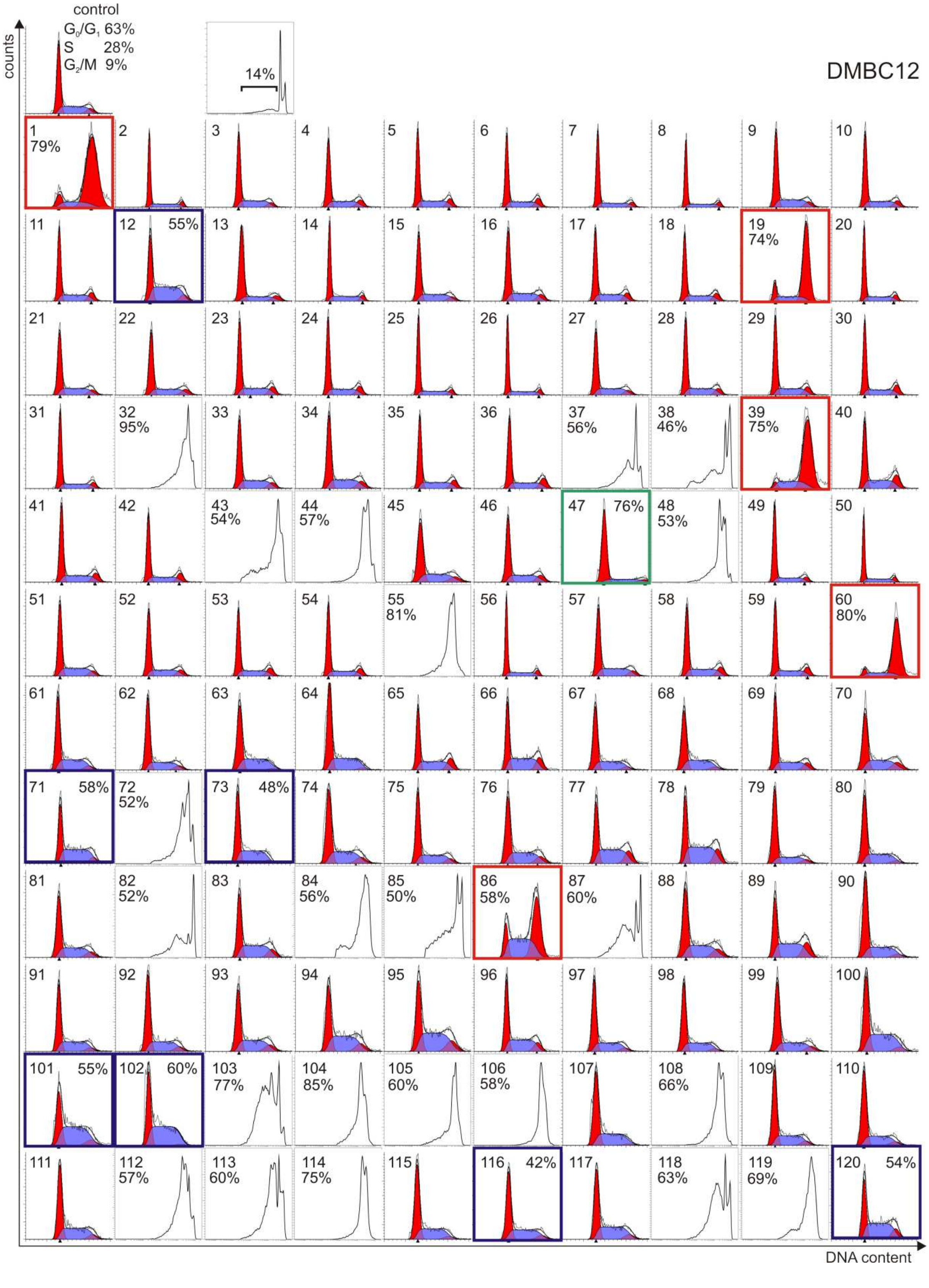

Figure S3B

Effects of lower concentrations for the most cytotoxic compounds or of longer exposure for compounds that were ineffective at 30 h.

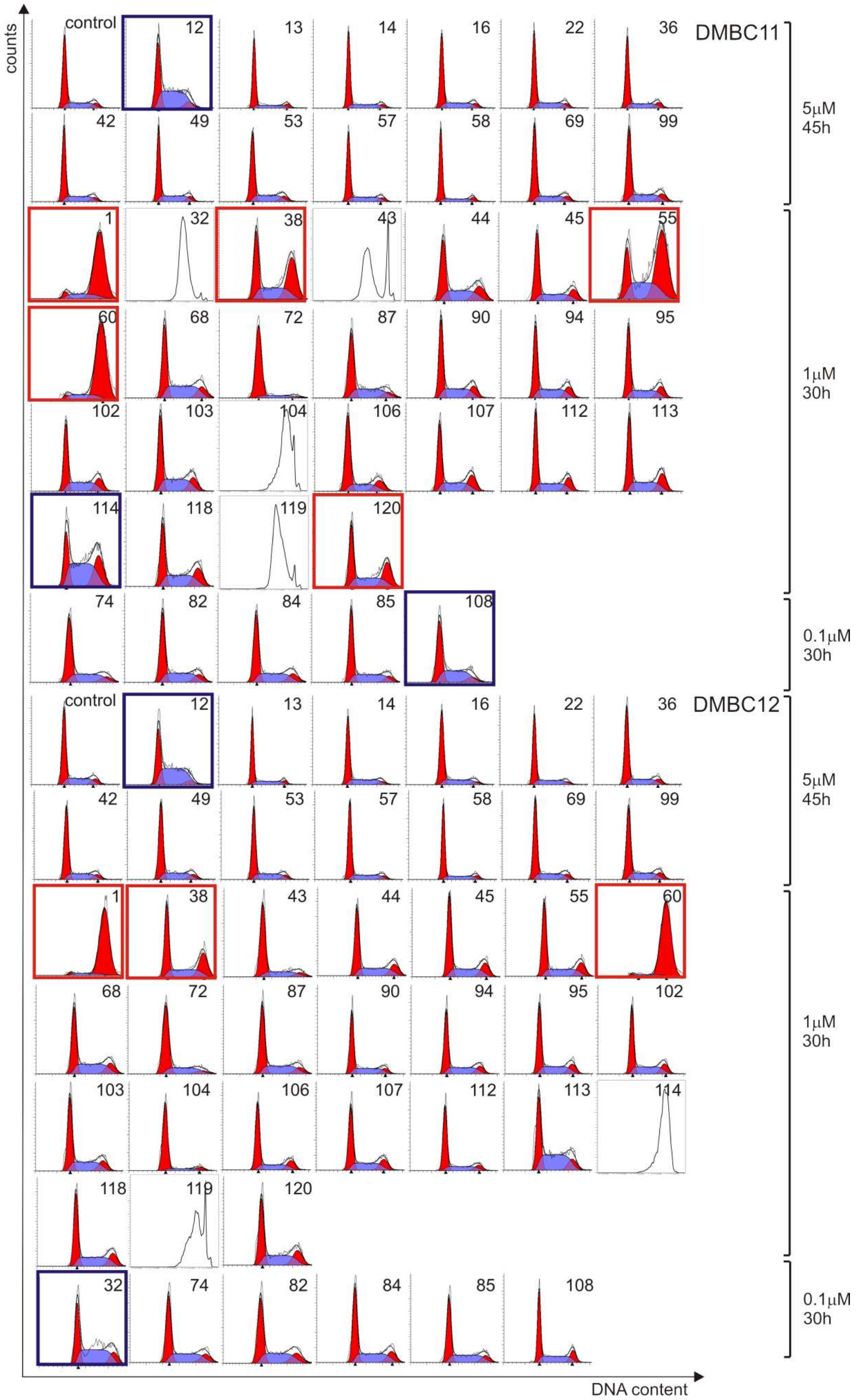

Figure S4

The influence of natural compounds used at a single concentration of 5 µM on the clonogenic growth of melanoma cells. Cells were incubated in drug-containing medium for 4 h and then they were grown on agar for 14 days in drug-free medium. Cell colonies were stained and counted. Anti-clonogenic activity was expressed as percentage of control treated with vehicle (0.05% DMSO). At least two independent experiments were performed in duplicates.

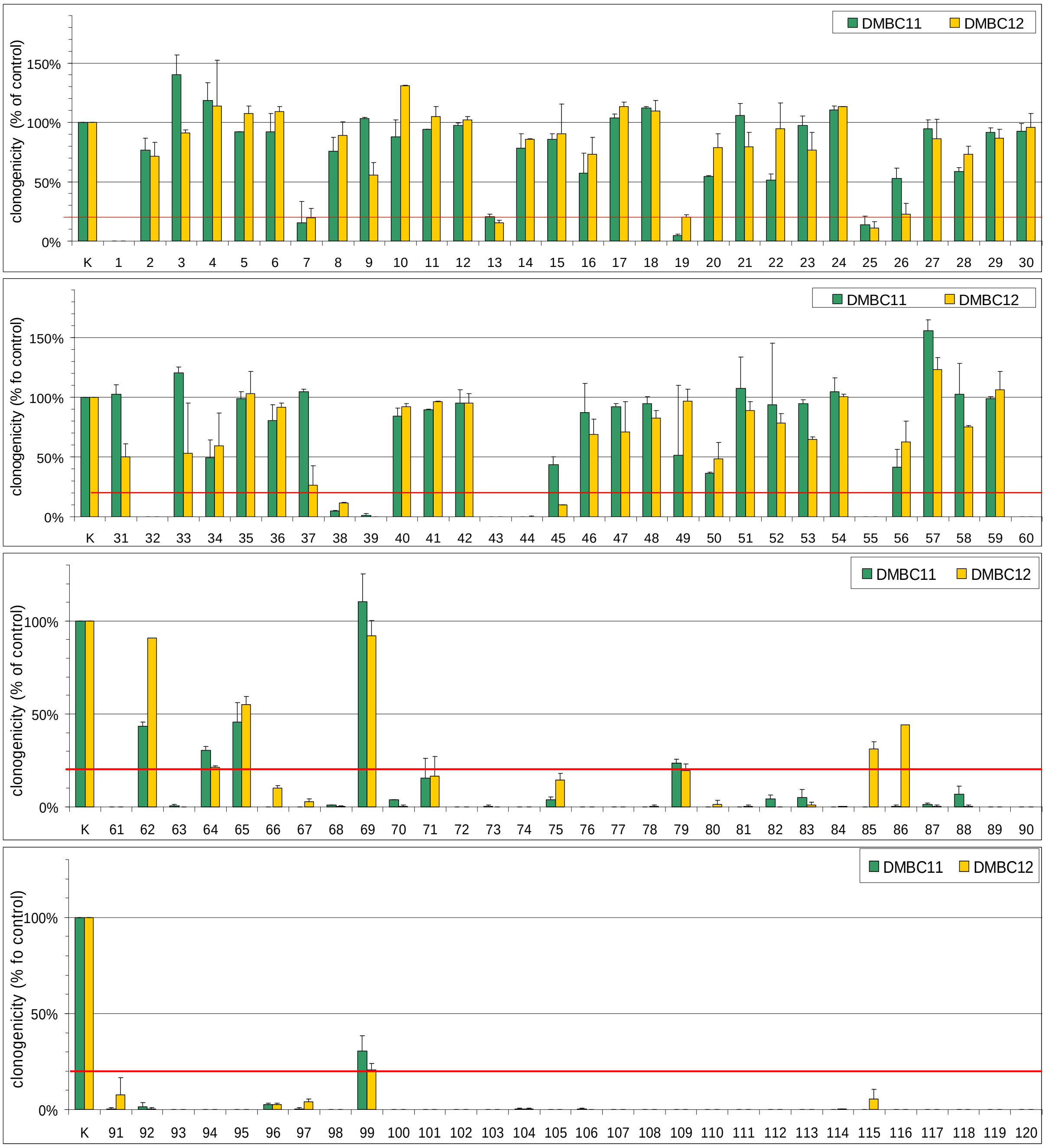

Table S2. Viability assessed in six different melanoma cell lines after 45 h of treatment with selected drugs at indicated concentration

viability at 1 µM

	DMBC:	2	8	9	10	11	12
60	maytansine	16% ± 5%	55% ± 4%	35% ± 2%	15% ± 1%	22% ± 4%	17% ± 3%
32	streptonigrin	2% ± 1%	3% ± 0%	2% ± 1%	1% ± 0%	4% ± 1%	3% ± 0%
108	toyocamycin	1% ± 0%	2% ± 1%	1% ± 1%	1% ± 1%	1% ± 0%	1% ± 1%
1	colchicine	24% ± 4%	52% ± 13%	36% ± 5%	21% ± 1%	28% ± 7%	21% ± 0%
102	echinomycin A	2% ± 0%	1% ± 1%	2% ± 3%	0% ± 0%	1% ± 1%	1% ± 1%
72	geldanamycin analog	10% ± 4%	4% ± 2%	13% ± 3%	16% ± 2%	16% ± 7%	8% ± 3%
104	didemnin B	1% ± 0%	1% ± 1%	9% ± 5%	2% ± 1%	3% ± 2%	2% ± 1%
119	tubulosine	3% ± 0%	9% ± 7%	4% ± 2%	1% ± 0%	2% ± 1%	1% ± 1%
74	nanaomycin	15% ± 9%	19% ± 1%	14% ± 1%	2% ± 1%	1% ± 1%	2% ± 1%
106	tetrocarcin A	24% ± 6%	4% ± 2%	17% ± 11%	9% ± 6%	9% ± 7%	10% ± 6%
86	rhizoxin	15% ± 2%	34% ± 18%	22% ± 10%	18% ± 9%	17% ± 12%	14% ± 6%
39	vincristine sulfate	17% ± 1%	29% ± 11%	20% ± 4%	19% ± 2%	16% ± 5%	22% ± 6%
114	illudin M	17% ± 8%	35% ± 19%	5% ± 4%	1% ± 0%	10% ± 5%	4% ± 3%
109	cucurbitacine E	9% ± 2%	35% ± 26%	11% ± 1%	12% ± 6%	8% ± 6%	8% ± 2%
84	bactobolin	12% ± 4%	61% ± 1%	30% ± 5%	12% ± 2%	10% ± 4%	11% ± 4%
44	helenalin	61% ± 12%	90% ± 11%	54% ± 15%	17% ± 6%	22% ± 4%	30% ± 7%
82	cytochalasin H	34% ± 11%	8% ± 1%	39% ± 16%	28% ± 18%	56% ± 11%	43% ± 5%
43	daunorubicin	56% ± 7%	77% ± 22%	20% ± 4%	27% ± 18%	29% ± 20%	37% ± 15%
113	4-ipomeanol	59% ± 1%	81% ± 15%	70% ± 1%	44% ± 14%	61% ± 7%	39% ± 1%
87	hispanolone	53% ± 35%	56% ± 31%	85% ± 13%	69% ± 23%	45% ± 25%	43% ± 18%
110	geldanamycin	76% ± 28%	85% ± 9%	86% ± 11%	48% ± 6%	50% ± 35%	58% ± 26%
112	bryostatin 1	61% ± 6%	80% ± 1%	68% ± 13%	57% ± 4%	74% ± 1%	52% ± 10%
89	croton factor F1	95% ± 19%	92% ± 18%	75% ± 16%	47% ± 16%	81% ± 9%	57% ± 18%
38	imidazoquinoline	71% ± 6%	64% ± 1%	75% ± 8%	86% ± 15%	89% ± 1%	89% ± 6%
68	crassin	106% ± 15%	108% ± 6%	90% ± 5%	67% ± 7%	70% ± 7%	67% ± 9%
81	helenin	94% ± 15%	126% ± 2%	76% ± 7%	61% ± 6%	57% ± 6%	70% ± 1%
107	siomycin A	81% ± 8%	76% ± 5%	88% ± 3%	63% ± 21%	89% ± 4%	67% ± 12%
85	baccatin III	76% ± 15%	89% ± 14%	83% ± 4%	76% ± 15%	60% ± 11%	69% ± 6%
101	michellamine B	89% ± 7%	83% ± 15%	90% ± 7%	77% ± 13%	96% ± 0%	72% ± 18%
103	valinomycin	74% ± 29%	79% ± 29%	80% ± 1%	94% ± 8%	95% ± 4%	96% ± 4%
100	pentoxifylline	95% ± 2%	90% ± 7%	98% ± 2%	85% ± 9%	98% ± 1%	86% ± 9%
118	fumitremorgin C	93% ± 16%	104% ± 22%	102% ± 1%	94% ± 2%	100% ± 0%	101% ± 4%
120	fumagillin	96% ± 10%	122% ± 28%	94% ± 4%	84% ± 7%	90% ± 6%	88% ± 4%
95	nordracorubin	82% ± 19%	87% ± 7%	99% ± 1%	96% ± 0%	99% ± 0%	90% ± 10%
94	confertofoline	93% ± 4%	77% ± 5%	101% ± 1%	96% ± 4%	100% ± 2%	95% ± 6%
45	parthenin	94% ± 4%	92% ± 21%	92% ± 1%	81% ± 17%	88% ± 7%	95% ± 4%
55	teniposide	90% ± 21%	80% ± 14%	90% ± 14%	90% ± 13%	88% ± 12%	93% ± 13%
63	fastigilin	108% ± 25%	121% ± 1%	91% ± 6%	84% ± 9%	84% ± 1%	87% ± 1%
19	rotenone	90% ± 10%	109% ± 24%	96% ± 2%	91% ± 0%	88% ± 4%	90% ± 1%
78	physalin B	107% ± 18%	110% ± 4%	96% ± 1%	91% ± 20%	92% ± 5%	91% ± 1%
111	Wortmannin	78% ± 4%	123% ± 14%	94% ± 1%	79% ± 6%	93% ± 1%	94% ± 3%
83	lonchocarpic acid	95% ± 4%	99% ± 13%	88% ± 1%	82% ± 28%	90% ± 12%	93% ± 3%
90	chaetochromin	88% ± 3%	99% ± 13%	99% ± 1%	100% ± 3%	84% ± 8%	90% ± 14%
116	castanospermine	94% ± 5%	96% ± 35%	103% ± 3%	96% ± 6%	101% ± 1%	92% ± 14%
115	pleurotine	87% ± 16%	114% ± 2%	96% ± 2%	93% ± 21%	95% ± 13%	96% ± 3%

viability at 0.1 µM

	DMBC:	2	8	9	10	11	12
60	maytansine	18% ± 4%	52% ± 7%	37% ± 5%	19% ± 5%	28% ± 8%	21% ± 4%
32	streptonigrin	50% ± 14%	33% ± 14%	14% ± 6%	20% ± 7%	28% ± 8%	37% ± 5%
108	toyocamycin	4% ± 3%	16% ± 11%	5% ± 3%	1% ± 1%	1% ± 1%	2% ± 1
1	colchicine	27% ± 4%	50% ± 11%	42% ± 8%	24% ± 5%	37% ± 4%	23% ± 1%
102	echinomycin A	5% ± 4%	2% ± 1%	3% ± 2%	1% ± 0%	3% ± 3%	2% ± 1%
72	geldanamycin analog	76% ± 2%	30% ± 20%	27% ± 1	38% ± 20%	21% ± 10%	36% ± 22%
104	didemnin B	74% ± 31%	99% ± 14%	79% ± 22%	58% ± 41%	64% ± 33%	66% ± 29%
119	tubulosine	93% ± 8%	97% ± 4%	104% ± 4%	105% ± 4%	104% ± 1%	105% ± 4%
74	nanaomycin	99% ± 1%	102% ± 1%	99% ± 0%	79% ± 16%	84% ± 0%	79% ± 7%
106	tetrocarcin A	83% ± 8%	95% ± 1%	100% ± 1%	98% ± 5%	98% ± 4%	97% ± 1%
86	rhizoxin	52% ± 42%	56% ± 33%	51% ± 33%	41% ± 33%	22% ± 13%	25% ± 24%
39	vincristine sulfate	88% ± 2%	34% ± 1%	82% ± 5%	84% ± 1%	73% ± 6%	64% ± 4%
114	illudin M	96% ± 11%	84% ± 10%	103% ± 0%	96% ± 6%	99% ± 3%	90% ± 3%
109	cucurbitacine E	94% ± 18%	80% ± 11%	90% ± 4%	89% ± 8%	87% ± 16%	88% ± 12%
84	bactobolin	96% ± 13%	93% ± 12%	91% ± 2%	84% ± 20%	81% ± 3%	96% ± 4%
44	helenalin	89% ± 4%	96% ± 25%	102% ± 2%	90% ± 14%	98% ± 5%	97% ± 7%
82	cytochalasin H	99% ± 1%	96% ± 9%	87% ± 6%	42% ± 21%	77% ± 6%	68% ± 4%
43	daunorubicin	95% ± 12%	83% ± 16%	97% ± 9%	102% ± 5%	99% ± 2%	98% ± 4%
113	4-ipomeanol	87% ± 2%	93% ± 10%	99% ± 1%	101% ± 4%	99% ± 1%	97% ± 1%
87	hispanolone	106% ± 9%	101% ± 2%	106% ± 1%	107% ± 14%	104% ± 6%	104% ± 0%
110	geldanamycin	103% ± 7%	101% ± 4%	107% ± 4%	116% ± 29%	103% ± 8%	107% ± 5%
112	bryostatin 1	95% ± 1%	98% ± 4%	84% ± 1%	80% ± 1%	87% ± 7%	75% ± 10%
89	croton factor F1	87% ± 9%	97% ± 4%	87% ± 14%	69% ± 12%	93% ± 9%	68% ± 9%
38	imidazoquinoline	77% ± 6%	112% ± 2%	94% ± 1%	85% ± 5%	93% ± 1%	96% ± 2%
68	crassin	112% ± 10%	102% ± 2%	103% ± 6%	93% ± 21%	98% ± 3%	105% ± 2%

81	helenin	97% ± 8%	116% ± 6%	92% ± 9%	87% ± 13%	77% ± 16%	92% ± 2%
107	siomycin A	88% ± 1%	93% ± 6%	96% ± 3%	95% ± 6%	98% ± 1%	102% ± 7%
85	baccatin III	108% ± 2%	100% ± 16%	91% ± 16%	93% ± 15%	89% ± 3%	101% ± 3%
101	michellamine B	93% ± 3%	99% ± 8%	104% ± 4%	107% ± 1%	105% ± 1%	104% ± 6%
103	valinomycin	82% ± 11%	105% ± 13%	105% ± 4%	105% ± 1%	104% ± 1%	103% ± 8%
100	pentoxifylline	97% ± 8%	99% ± 13%	105% ± 4%	106% ± 1%	105% ± 1%	106% ± 8%
118	fumitremorgin C	95% ± 14%	103% ± 4%	105% ± 0%	106% ± 6%	107% ± 1%	106% ± 3%
120	fumagillin	100% ± 8%	114% ± 12%	100% ± 3%	105% ± 4%	98% ± 3%	93% ± 8%
95	nordracorubin	91% ± 13%	94% ± 4%	105% ± 4%	105% ± 3%	105% ± 1%	101% ± 4%
94	confertofoline	98% ± 5%	78% ± 16%	105% ± 4%	104% ± 2%	105% ± 0%	105% ± 1%
45	parthenin	101% ± 10%	86% ± 8%	100% ± 2%	103% ± 5%	101% ± 0%	101% ± 1%
55	teniposide	86% ± 8%	93% ± 3%	89% ± 13%	96% ± 4%	93% ± 3%	99% ± 4%
63	fastigilin	108% ± 14%	115% ± 4%	96% ± 10%	83% ± 21%	90% ± 10%	99% ± 9%
19	rotenone	95% ± 6%	115% ± 9%	99% ± 0%	98% ± 6%	100% ± 4%	101% ± 6%
78	physalin B	106% ± 6%	108% ± 1%	102% ± 4%	95% ± 18%	96% ± 9%	104% ± 4%
111	wortmannin	82% ± 4%	127% ± 4%	101% ± 1%	89% ± 3%	99% ± 1%	102% ± 1%
83	lonchocarpic acid	104% ± 9%	101% ± 3%	100% ± 3%	84% ± 24%	97% ± 10%	101% ± 6%
90	chaetochromin	89% ± 8%	102% ± 5%	100% ± 1%	100% ± 2%	102% ± 1%	102% ± 1%
116	castanospermine	95% ± 1%	99% ± 22%	108% ± 4%	102% ± 7%	106% ± 0%	103% ± 4%
115	pleurotine	99% ± 4%	104% ± 1%	105% ± 1%	110% ± 25%	98% ± 21%	98% ± 6%

viability at 0.01 µM

	DMBC:	2	8	9	10	11	12
60	maytansine	10% ± 3%	34% ± 6%	28% ± 3%	15% ± 3%	19% ± 4%	15% ± 4%
32	streptonigrin	65% ± 15%	67% ± 6%	47% ± 10%	69% ± 8%	71% ± 8%	67% ± 6%
108	toyocamycin	101% ± 26%	95% ± 1%	103% ± 1%	101% ± 8%	105% ± 3%	97% ± 0%
1	colchicine	105% ± 15%	101% ± 8%	97% ± 5%	98% ± 2%	94% ± 3%	94% ± 12%
102	echinomycin A	87% ± 13%	86% ± 3%	78% ± 6%	97% ± 0%	85% ± 8%	106% ± 2%
104	didemnin B	106% ± 19%	92% ± 5%	105% ± 4%	103% ± 8%	106% ± 8%	106% ± 5%
86	rhizoxin	102% ± 17%	96% ± 6%	103% ± 9%	105% ± 8%	104% ± 8%	104% ± 0%
39	vincristine sulfate	107% ± 1%	106% ± 7%	105% ± 16%	102% ± 1%	98% ± 0%	97% ± 3%
109	cucurbitacine	105% ± 17%	100% ± 7%	105% ± 0%	104% ± 8%	108% ± 5%	103% ± 7%
43	daunorubicin	96% ± 13%	97% ± 8%	100% ± 9%	102% ± 5%	98% ± 3%	98% ± 3%
110	geldanamycin	95% ± 4%	97% ± 7%	101% ± 6%	114% ± 20%	104% ± 0%	99% ± 8%

viability at 0.001 µM

	DMBC:	2	8	9	10	11	12
60	maytansine	102% ± 20%	95% ± 5%	100% ± 9%	99% ± 6%	96% ± 9%	97% ± 2%
32	streptonigrin	91% ± 26%	87% ± 1%	95% ± 3%	98% ± 7%	96% ± 2%	93% ± 2%
108	toyocamycin	101% ± 26%	89% ± 4%	99% ± 2%	100% ± 8%	100% ± 3%	100% ± 1%
1	colchicine	98% ± 12%	107% ± 10%	92% ± 1%	89% ± 3%	87% ± 5%	91% ± 6%
39	vincristine sulfate	107% ± 6%	102% ± 2%	103% ± 7%	102% ± 1%	100% ± 4%	101% ± 2%
43	daunorubicin	99% ± 7%	97% ± 0%	103% ± 11%	102% ± 7%	94% ± 3%	97% ± 6%

Viability was measured by flow cytometry after PI staining in six different melanoma cell lines DMBC2, DMBC8, DMBC9, DMBC10, DMBC11 and DMBC12.
Data expressed as % of control are means ± SD of two independent experiments conducted in triplicates.

Figure S5.
Dose-response curves prepared for compounds exerting anti-clonogenic and/or cytotoxic potentials. Blue curves, anti-clonogenic activity; black curves, cytotoxic activity; DMBC11 (filled square) and DMBC12 (open square) cell lines.

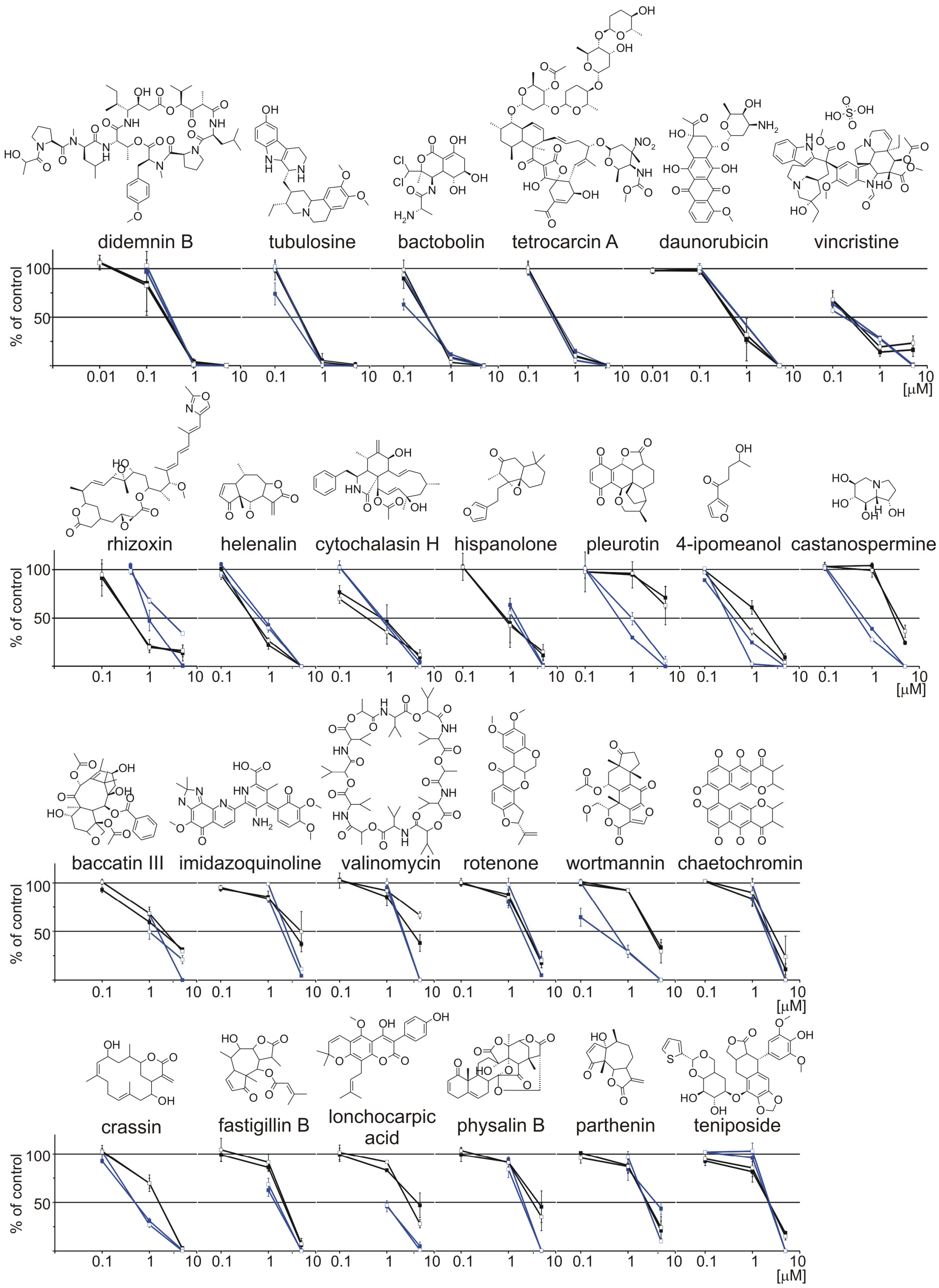

Table S3. Activity profiles of natural compounds selected in this study prepared based on a literature search. Only the main biological activities of compounds are included.

Compound and its source	Main biological activities
**Nanaomycin A**<br>*Streptomyces rosa*	selective inhibitor of DNMT3B (DNA methyltransferase 3B) that reactivates the expression of silenced tumor suppressor gene *RASSF1A* in human cancer cells [1];
**Illudin M**<br>*Omphalotus illudens*	alkylating agent of DNA, RNA and proteins [2];
**Geldanamycin**<br>*Streptomyces hygroscopicus*	inhibitor of Hsp90 (heat shock protein 90) [3];
**Bryostatin 1**<br>*Bugula neritina*	highly potent activator of PKC (protein kinase C) [4];<br>ligand for TLR4 (Toll-like receptor 4) triggering NF-κB (nuclear factor-kappa B) activity and the expression of interleukins (IL-5, Il-6, IL-10) and chemokines: RANTES (regulated on activation normal T cell expressed and secreted) and MIP1-α (macrophage inflammatory protein 1α) [5];<br>activator of STAT1 (signal transducer and activator of transcription 1) activity through an IFNγ (interferon gamma) autocrine loop [6];<br>enhancer of CD4+ T cell-mediated recognition of melanoma cells, inducer of the expression of costimulatory molecules (CD80 and CD86) in melanoma cells prolonging immune response, inducer of melanoma cell differentiation [7];
**Siomycin A**<br>*Streptomyces sioyaensis*	inhibitor of the oncogenic transcription factor FoxM1 (forkhead box M1) and selective inducer of apoptosis in transformed cells [8,9];<br>proteasome inhibitor stabilizing the expression of p21, Mcl-1, p53 and Hdm-2 [10];<br>inhibitor of MELK (maternal embryonic leucine zipper kinase) [11];
**Fumitremorgin C**<br>*Aspergillus elongatus, Aspergillus fumigatus*	inhibitor of ABCG2/BCRP (breast cancer resistance protein) [12];<br>inhibitor of the AKT pathway [13];
**Fumagillin**<br>*Aspergillus fumigatus*	inhibitor of MetAP-2 (methionine aminopetidase-2) [14];<br>anti-angiogenic agent reducing the expression of cyclin E2, ALCAM (activated leukocyte cell adhesion molecule) and ICAM-1 (intercellular adhesion molecule-1) [15];<br>inhibitor of FGFR1 (fibroblast growth factor receptor 1) [16];
**Michellamine B**<br>*Ancistrocladus korupensis*	inhibitor of HIV (human immunodeficiency virus) reverse transcriptase and human DNA polymerases α and β [17];<br>inhibitor of PKC [18];<br>mitochondria-protective agent against adenosine diphosphate- and Fe^{2+}-induced lipid peroxidation [19];

**Pentoxifylline**	competitive non-specific phosphodiesterase inhibitor [20];<br>activator of PKA (protein kinase A), inhibitor of TNF-α (tumor necrosis factor alpha) production [21,22];<br>inducer of lipid peroxidation increasing the activity of glutathione-S-transferase and leading to glutathione depletion [23];<br>inhibitor of MMP-2 and MMP-9 (metalloproteinase-2 and -9) secretion [24];<br>inducer of apoptosis related to up-regulation of DR4 and DR5 (death receptor-4 and -5) expression on cell surface, and down-regulation of the expression of anti-apoptotic proteins [25];
**Croton Factor F1**	unidentified
**Helenin**<br>*Helenium autumnale*	unidentified
**Nordracorubin**<br>*Daemonorops draco*	unidentified
**Confertifoline**<br>*Polygonum hydropiper*	antimicrobial agent [26];
**Wortmannin**<br>*Penicillium funiculosum*	inhibitor of MAPK (mitogen-activated protein kinase) [27];<br>inhibitor of PI3K (phosphoinositide-3-kinase) [28];
**4-Ipomeanol**	unidentified
**Crassin**<br>*Pseudoplexaura porosa,*<br>*Pseudoplexaura flagellosa,*<br>*Pseudoplexaura wagenaari,*<br>*Pseudoplexaura crucis*	inhibitor of allogeneic leukocyte reaction as well as antigen-specific activation of CD4+ T cells by bone marrow-derived dendritic cells [29];
**Castanospermine**<br>*Castanospermum australe*	inhibitor of selected glucosidase enzymes [30];
**Lonchocarpic Acid**<br>*Lonchocarpus*	unidentified
**Pleurotin**<br>*Pleurotus griseus*	inhibitor of HIF-1α (hypoxia-induced factor-1alpha) and VEGF (vascular endothelial growth factor) expression [31];
**Maytansine**<br>*Maytenus ovatus*	inhibitor of the microtubule assembly by binding to tubulin at or near the rhizoxin-binding site [32];
**Streptonigrin**<br>*Streptomyces flocculus*	inhibitor of DNA/RNA synthesis and topoisomerase II [33];<br>inhibitor of β-catenin/TCF signaling [34];
**Toyocamycin**<br>*Streptomyces toyocaensis*	inhibitor of RNA synthesis and splicing, ribosome maturation and function [35,36,37];
**Colchicine**<br>*Colchicum autumnale*	inhibitor of microtubule polymerization by binding to tubulin [38];<br>inducer of apoptosis accompanied by loss of mitochondrial membrane potential, activator of caspase-3 and -9, and inhibitor of Bcl-2 (B-cell leukemia/lymphoma 2) expression [39];
**Echinomycin A**<br>*Streptomyces echinatus*<br>*Streptomyces lasalienis*	DNA intercalator targeting HIF-1α [40];<br>suppressor of NOTCH1, MYC, AKT, mTOR signaling [41];

**Cucurbitacin E**<br>*Cucurbitaceae*	suppressor of NF-κB activity [42];<br>inducer of caspase-3-mediated apoptosis [43];<br>inhibitor of VEGFR2 (vascular endothelial growth factor receptor 2)-mediated Jak-STAT3 and MAPK signaling pathways [44];
**Didemnin B**<br>*Trididemnum solidum*	inhibitor of DNA and protein synthesis [45];
**Tubulosine**<br>*Pogonopus tubulosus*	inhibitor of peptide chain elongation during protein synthesis [46];
**Tetrocarcin A**<br>*Micromonospora*	inhibitor of the anti-apoptotic function of Bcl-2 [47];<br>activator of intrinsic pathway of apoptosis, inducer of Hsp70 and Hsp110 expression [48];
**Vincristine**<br>*Catharanthus roseus*	inhibitor of microtubule assembly by binding to tubulin dimmers [49];
**Bactobolin**<br>*Pseudoinonas*	unidentified
**Helenalin**<br>*Arnica montana,*<br>*Arnica chamissonis foliosa*	inhibitor of NF-κB [50];<br>selective inhibitor of telomerase [51];
**Cytochalasin H**	inhibitor of cytoskeletal reorganization [52];
**Daunorubicin** (43)<br>*Streptomyces peucetius*	inhibitor of topoisomerase II [53];
**Hispanolone**	unidentified
**Geldanamycin analog**<br>*Streptomyces hygroscopicus*	inhibitor of Hsp90 [54];<br>inducer of MITF (microphthalmia-associated transcription factor) expression [55];<br>inhibitor of cytochrome P450 enzymes: CYP3A4/5 and CYP2C19 [56];
**Rhizoxin**<br>*Rhizopus microsporus*	inhibitor of cell division through binding to β-tubulin and disrupting microtubule formation [57];
**Baccatin III**<br>*Taxus brevifolia*<br>*Fusarium solani*	inhibitor of spindle function, inducer of caspase-10-dependent apoptosis [58];
**Imidazoquinoline**	inducer of TLR7-mediated immune cell activation [59];
**Rotenone**<br>*Lonchocarpus nicou*<br>*Derris elliptica*	inhibitor of the transfer of electrons from iron-sulfur centers in complex I to ubiquinone in electron transport chain [60];
**Chaetochromin**<br>*Chaetomium spp.*	inhibitor of DNA, RNA and protein synthesis [61];
**Fastigilin B**<br>*Baileya multiradiata*	unidentified
**Physalin B**<br>*Physalis alkekengi,*<br>*Physalis angulata,*<br>*Physalis lancifolia*	inhibitor of the ubiquitin-proteasome pathway [62];<br>inducer of apoptosis *via* JNK (c-Jun N-terminal kinase) and/or ERK (extracellular signal-regulated kinase) activation, decreases androgen receptor expression [63];<br>inducer of mitochondria-mediated apoptosis pathway [64];
**Parthenin**<br>*Parthenium hysterophorus*	modulator of oxidative phosphorylation [65];
**Teniposide**	inhibitor of DNA synthesis that forms a complex with topoisomerase II and DNA [66];

**Valinomycin**<br>*Streptomyces tsusimaensis*<br>*Streptomyces fulvissimus*	natural ionophore with potassium-specific transporter activity [67].

References to Table S3

1. Kuck D, Caulfield T, Lyko F, Medina-Franco JL (2010) Nanaomycin A selectively inhibits DNMT3B and reactivates silenced tumor suppressor genes in human cancer cells. Mol Cancer Ther 9: 3015-3023.

2. McMorris TC, Kelner MJ, Wang W, Moon S, Taetle R (1990) On the mechanism of toxicity of illudins: the role of glutathione. Chem Res Toxicol 3: 574-579.

3. Schulte TW, Akinaga S, Soga S, Sullivan W, Stensgard B, et al. (1998) Antibiotic radicicol binds to the N-terminal domain of Hsp90 and shares important biologic activities with geldanamycin. Cell Stress Chaperones 3: 100-108.

4. Yi P, Schrott L, Castor TP, Alexander JS (2012) Bryostatin-1 vs. TPPB: dose-dependent APP processing and PKC-α, -δ, and -ε isoform activation in SH-SY5Y neuronal cells. J Mol Neurosci 48: 234-244.

5. Ariza ME, Ramakrishnan R, Singh NP, Chauhan A, Nagarkatti PS, et al. (2011) Bryostatin-1, a naturally occurring antineoplastic agent, acts as a Toll-like receptor 4 (TLR-4) ligand and induces unique cytokines and chemokines in dendritic cells. J Biol Chem 286: 24-34.

6. Battle TE, Frank DA (2003) STAT1 mediates differentiation of chronic lymphocytic leukemia cells in response to Bryostatin 1. Blood 102: 3016-3024.

7. Zhao D, Amria S, Hossain A, Sundaram K, Komlosi P, et al. (2011) Enhancement of HLA class II-restricted CD4+ T cell recognition of human melanoma cells following treatment with bryostatin-1. Cell Immunol 271: 392-400.

8. Radhakrishnan SK, Bhat UG, Hughes DE, Wang IC, Costa RH, et al. (2006) Identification of a chemical inhibitor of the oncogenic transcription factor forkhead box M1. Cancer Res 66: 9731-9735.

9. Bhat UG, Halasi M, Gartel AL (2009) Thiazole antibiotics target FoxM1 and induce apoptosis in human cancer cells. PLoS One 4: e5592.

10. Bhat UG, Halasi M, Gartel AL (2009) FoxM1 is a general target for proteasome inhibitors. PLoS One 4: e6593.

11. Kuner R, Fälth M, Pressinotti NC, Brase JC, Puig SB, et al. (2013) The maternal embryonic leucine zipper kinase (MELK) is upregulated in high-grade prostate cancer. J Mol Med (Berl) 91: 237-248.

12. Rabindran SK, Ross DD, Doyle LA, Yang W, Greenberger LM (2000) Fumitremorgin C reverses multidrug resistance in cells transfected with the breast cancer resistance protein. Cancer Res 60: 47-50.

13. Wang L, Sasai K, Akagi T, Tanaka S (2008) Establishment of a luciferase assay-based screening system: fumitremorgin C selectively inhibits cellular proliferation of immortalized astrocytes expressing an active form of AKT. Biochem Biophys Res Commun 373: 392-396.

14. Sin N, Meng L, Wang MQ, Wen JJ, Bornmann WG, et al. (1997) The anti-angiogenic agent fumagillin covalently binds and inhibits the methionine aminopeptidase, MetAP-2. Proc Natl Acad Sci U S A 94: 6099-6103.

15. Hou L, Mori D, Takase Y, Meihua P, Kai K, et al. (2009) Fumagillin inhibits colorectal cancer growth and metastasis in mice: in vivo and in vitro study of anti-angiogenesis. Pathol Int 59: 448-461.

16. Chen GJ, Weylie B, Hu C, Zhu J, Forough R (2007) FGFR1/PI3K/AKT signaling pathway is a novel target for antiangiogenic effects of the cancer drug fumagillin (TNP-470). J Cell Biochem 101: 1492-1504.

17. McMahon JB, Currens MJ, Gulakowski RJ, Buckheit RW Jr, Lackman-Smith C, et al. (1995) Michellamine B, a novel plant alkaloid, inhibits human immunodeficiency virus-induced cell killing by at least two distinct mechanisms. Antimicrob Agents Chemother 39: 484-488.

18. White EL, Chao WR, Ross LJ, Borhani DW, Hobbs PD, et al. (1999) Michellamine alkaloids inhibit protein kinase C. Arch Biochem Biophys 365: 25-30.

19. White EL, Ross LJ, Hobbs PD, Upender V, Dawson MI (1999) Antioxidant activity of michellamine alkaloids. Anticancer Res 19: 1033-1035.

20. Essayan DM (2001) Cyclic nucleotide phosphodiesterases. J Allergy Clin Immunol 108: 671–680.

21. Marques LJ, Zheng L, Poulakis N, Guzman J, Costabel U (1999) Pentoxifylline inhibits TNF-alpha production from human alveolar macrophages. Am J Respir Crit Care Med 159: 508–511.

22. Deree J, Martins JO, Melbostad H, Loomis WH, Coimbra R (2008) Insights into the regulation of TNF-α production in human mononuclear cells: the effects of non-specific phosphodiesterase inhibition. Clinics (Sao Paulo) 63: 321–328.

23. Shukla V, Gude RP (2003) Potentiation of antimetastatic activity of pentoxifylline in B16F10 and B16F1 melanoma cells through inhibition of glutathione content. Cancer Biother Radiopharm 18: 559-564.

24. Dua P, Gude RP (2006) Antiproliferative and antiproteolytic activity of pentoxifylline in cultures of B16F10 melanoma cells. Cancer Chemother Pharmacol 58: 195-202.

25. Gahlot S, Khan MA, Rishi L, Majumdar S (2010) Pentoxifylline augments TRAIL/Apo2L mediated apoptosis in cutaneous T cell lymphoma (HuT-78 and MyLa) by modulating the expression of antiapoptotic proteins and death receptors. Biochem Pharmacol 80: 1650-1661.

26. Duraipandiyan V, Indwar F, Ignacimuthu S (2010) Antimicrobial activity of confertifolin from Polygonum hydropiper. Pharm Biol 48: 187-190.

27. Ferby IM, Waga I, Hoshino M, Kume K, Shimizu T (1996) Wortmannin inhibits mitogen-activated protein kinase activation by platelet-activating factor through a mechanism independent of p85/p110-type phosphatidylinositol 3-kinase. J Biol Chem 271: 11684-11688.

28. Wymann MP, Bulgarelli-Leva G, Zvelebil MJ, Pirola L, Vanhaesebroeck B, et al. (1996) Wortmannin inactivates phosphoinositide 3-kinase by covalent modification of

Lys-802, a residue involved in the phosphate transfer reaction. Mol Cell Biol 16: 1722-1733.

29. Matsushima H, Tanaka H, Mizumoto N, Takashima A (2009) Identification of crassin acetate as a new immunosuppressant triggering heme oxygenase-1 expression in dendritic cells. Blood 114: 64-73.

30. Saul R, Ghidoni JJ, Molyneux RJ, Elbein AD (1985) Castanospermine inhibits alpha-glucosidase activities and alters glycogen distribution in animals. Proc Natl Acad Sci U S A 82: 93–97.

31. Welsh SJ, Williams RR, Birmingham A, Newman DJ, Kirkpatrick DL, et al. (2003) The thioredoxin redox inhibitors 1-methylpropyl 2-imidazolyl disulfide and pleurotin inhibit hypoxia-induced factor 1alpha and vascular endothelial growth factor formation. Mol Cancer Ther 2: 235-243.

32. Cassady JM, Chan KK, Floss HG, Leistner E (2004) Recent developments in the maytansinoid antitumor agents. Chem Pharm Bull (Tokyo) 52: 1–26.

33. Bolzan AD, Bianchi MS (2001) Genotoxicity of streptonigrin: a review. Mutat Res 488: 25-37.

34. Park S, Chun S (2011) Streptonigrin inhibits β-Catenin/Tcf signaling and shows cytotoxicity in β-catenin-activated cells. Biochim Biophys Acta 1810: 1340-1345.

35. Suhadolnik RJ, Uematsu T, Uematsu H (1967) Toyocamycin: phosphorylation and incorporation into RNA and DNA and the biochemical properties of the triphosphate. Biochim Biophys Acta 149: 41-49.

36. Tavitian A, Uretsky SC, Acs G (1969) The effect of toyocamycin on cellular RNA synthesis. Biochim Biophys Acta. 179: 50-57.

37. Kiburu IN, LaRonde-LeBlanc N (2012) Interaction of Rio1 kinase with toyocamycin reveals a conformational switch that controls oligomeric state and catalytic activity. PLoS One 7: e37371.

38. Skoufias DA, Wilson L (1992) Mechanism of inhibition of microtubule polymerization by colchicine: inhibitory potencies of unliganded colchicine and tubulin-colchicine complexes. Biochemistry 31: 738-746.

39. Chen XM, Liu J, Wang T, Shang J (2012) Colchicine-induced apoptosis in human normal liver L-02 cells by mitochondrial mediated pathways. Toxicol In Vitro 26: 649-655.

40. Wang R, Zhou S, Li S (2011) Cancer therapeutic agents targeting hypoxia-inducible factor-1. Curr Med Chem 18: 3168-3189.

41. Yonekura S, Itoh M, Okuhashi Y, Takahashi Y, Ono A, et al. (2013) Effects of the HIF1 inhibitor, echinomycin, on growth and NOTCH signalling in leukaemia cells. Anticancer Res 33: 3099-3103.

42. Qiao J, Xu LH, He J, Ouyang DY, He XJ (2013) Cucurbitacin E exhibits anti-inflammatory effect in RAW 264.7 cells via suppression of NF-κB nuclear translocation. Inflamm Res 62: 461-469.

43. Lan T, Wang L, Xu Q, Liu W, Jin H, et al. (2013) Growth inhibitory effect of Cucrbitacin E on breast cancer cells. Int J Clin Exp Pathol 6: 1799-1805.

44. Chen X, Bao J, Guo J, Ding Q, Lu J, et al. (2012) Biological activities and potential molecular targets of cucurbitacins. Anti-Cancer Drugs 23: 777–787.

45. Li LH, Timmins LG, Wallace TL, Krueger WC, Prairie MD, et al. (1984) Mechanism of action of didemnin B, a depsipeptide from the sea. Cancer Lett 23: 279-288.

46. Carrasco L, Jimenez A, Vazquez D (1976) Specific inhibition of translocation by tubulosine in eukaryotic polysomes. Eur J Biochem 64: 1-5.

47. Nakashima T, Miura M, Hara M (2000) Tetrocarcin A inhibits mitochondrial functions of Bcl-2 and suppresses its anti-apoptotic activity. Cancer Res 60: 1229-1235.

48. Tinhofer I, Anether G, Senfter M, Pfaller K, Bernhard D, et al. (2002) Stressful death of T-ALL tumor cells after treatment with the anti-tumor agent Tetrocarcin-A. FASEB J 16: 1295-1297.

49. Lobert S, Vulevic B, Correia JJ (1996) Interaction of vinca alkaloids with tubulin: a comparison of vinblastine, vincristine, and vinorelbine. Biochemistry 35: 6806-6814.

50. Lyss G, Knorre A, Schmidt TJ, Pahl HL, Merfort I (1998) The anti-inflammatory sesquiterpene lactone helenalin inhibits the transcription factor NF-kappaB by directly targeting p65. J Biol Chem 273: 33508–33516.

51. Huang PR, Yeh YM, Wang TC (2005) Potent inhibition of human telomerase by helenalin. Cancer Lett 227: 169–174.

52. Natarajan P, May JA, Sanderson HM, Zabe M, Spangenberg P, et al. (2000) Effects of cytochalasin H, a potent inhibitor of cytoskeletal reorganisation, on platelet function. Platelets 11: 467-476.

53. Laurent G, Jaffrezou JP (2001) Signaling pathways activated by daunorubicin. Blood. 98: 913-924.

54. Toyomura K, Saito T, Emori S, Matsumoto I, Kato E, et al. (2012) Effects of Hsp90 inhibitors, geldanamycin and its analog, on ceramide metabolism and cytotoxicity in PC12 cells. J Toxicol Sci 37: 1049-1057.

55. van der Kraan AG, Chai RC, Singh PP, Lang BJ, Xu J, et al. (2013) HSP90 inhibitors enhance differentiation and MITF (microphthalmia transcription factor) activity in osteoclast progenitors. Biochem J 451: 235-244.

56. Gan J, Liu-Kreyche P, Humphreys WG (2012) In vitro assessment of cytochrome P450 inhibition and induction potential of tanespimycin and its major metabolite, 17-amino-17-demethoxygeldanamycin. Cancer Chemother Pharmacol 69: 51-56.

57. Takahashi M, Iwasaki S, Kobayashi H, Okuda S, Murai T, et al. (1987) Studies on macrocyclic lactone antibiotics. XI. Anti-mitotic and anti-tubulin activity of new antitumor antibiotics, rhizoxin and its homologues. J Antibiot 40: 66–72.

58. Chakravarthi BV, Sujay R, Kuriakose GC, Karande AA, Jayabaskaran C (2013) Inhibition of cancer cell proliferation and apoptosis-inducing activity of fungal taxol and its precursors baccatin III purified from endophytic Fusarium solani. Cancer Cell Int 13: 105.

59. Hemmi H, Kaisho T, Takeuchi O, Sato S, Sanjo H, et al. (2002) Small-antiviral compounds activate immune cells via the TLR7 MyD88-dependent signaling pathway. Nat Immunol 3: 196–200.

60. Hayes WJ (1991) Handbook on pesticides, Volume 1. Academic Press.

61. Koyama K, Ominato K, Natori S, Tashiro T, Tsuruo T (1988) Cytotoxicity and antitumor activities of fungal bis(naphtho-gamma-pyrone) derivatives. J Pharmacobiodyn 11: 630-635.

62. Vandenberghe I, Creancier L, Vispe S, Annereau JP, Barret JM, et al. (2008) Physalin B, a novel inhibitor of the ubiquitin-proteasome pathway, triggers NOXA-associated apoptosis. Biochem Pharmacol 76: 453-462.

63. Han H, Qiu L, Wang X, Qiu F, Wong Y, et al. (2011) Physalins A and B inhibit androgen-independent prostate cancer cell growth through activation of cell apoptosis and downregulation of androgen receptor expression. Biol Pharm Bull 34: 1584-1588.

64. Hsu CC, Wu YC, Farh L, Du YC, Tseng WK, et al. (2012) Physalin B from Physalis angulata triggers the NOXA-related apoptosis pathway of human melanoma A375 cells. Food Chem Toxicol 50: 619-624.

65. Narasimhan TR, Harindranath N, Kurup CK, Rao PV (1985) Effect of parthenin on mitochondrial oxidative phosphorylation. Biochem Int 11: 239-244.

66. Long BH (1992) Mechanisms of action of teniposide (VM-26) and comparison with etoposide (VP-16). Semin Oncol 19: 3-19.

67. Cammann K (1985) Ion-selective bulk membranes as models. Top Curr Chem 128: 219–258.
